# A novel immune-related gene signature stratifies prognosis and characterizes the tumor immune microenvironment in head and neck squamous cell carcinoma

**DOI:** 10.3389/fcell.2026.1756922

**Published:** 2026-04-08

**Authors:** Pian Li, Keling Pang, Zhigang Zhou, Chaolin Yang, Yi Chen, Yangjie Wu, Fujue Wang, Junyan He

**Affiliations:** 1 Department of Oncology, The First Affiliated Hospital, Hengyang Medical School, University of South China, Hengyang, Hunan, China; 2 Department of Radiation Oncology, The First Affiliated Hospital of Guangxi Medical University, Nanning, Guangxi, China; 3 Department of Oncology, Changde Hospital, Xiangya School of Medicine, Central South University (The First People’s Hospital of Changde City), Changde, Hunan, China; 4 Department of Oncology, The Affiliated Nanhua Hospital, Hengyang Medical School, University of South China, Hengyang, Hunan, China; 5 Department of Hematology, The First Affiliated Hospital, Hengyang Medical School, University of South China, Hengyang, Hunan, China; 6 State Key Laboratory of Biotherapy/Collaborative Innovation Center for Biotherapy, West China Hospital, Sichuan University, Chengdu, Sichuan, China

**Keywords:** head and neck squamous cell carcinoma (HNSCC), immune-related genes (IRGs), immunotherapy response, prognostic signature, PYGL, single-cell RNA sequencing (scRNA-seq), tumor microenvironment (TME)

## Abstract

**Background:**

Head and neck squamous cell carcinoma (HNSCC) exhibits heterogeneous responses to immunotherapy, primarily determined by the tumor immune microenvironment (TME). Accordingly, there is an urgent need for reliable biomarkers that can effectively distinguish between immune “hot” and “cold” tumors and predict prognosis and immunotherapy response.

**Methods:**

We integrated transcriptomic and clinical data from The Cancer Genome Atlas (TCGA) and Gene Expression Omnibus (GEO) cohorts to identify immune-related genes (IRGs) associated with TME phenotypes. Unsupervised clustering was used to define immune-hot and immune-cold subtypes. A six-gene prognostic signature (*PYGL*, *SFRP1*, *FGD3*, *OLR1*, *DUSP9*, and *MASP1*) was developed using Cox and Least Absolute Shrinkage and Selection Operator (LASSO) regression, which was validated across four cohorts and evaluated for its association with immune infiltration, tumor mutation burden (TMB), and immunotherapy response. The functional role of PYGL was assessed via siRNA knockdown in HNSCC cell lines.

**Results:**

The signature demonstrated good performance in stratifying patients into high- and low-risk groups, characterized by distinct survival, immune landscapes, and genomic mutation profiles. High-risk patients showed an immunosuppressive TME, higher TMB, and poorer response to immune checkpoint blockade. PYGL knockdown suppressed proliferation, migration, and clonogenicity. Transcriptomic analysis revealed downregulation of MYC/E2F targets and EMT pathways, alongside concurrent activation of interferon and immune response programs**.**

**Conclusion:**

This IRG-based signature offers a clinically translatable tool for prognostication and immunotherapy selection in HNSCC. PYGL represents a key oncogenic driver and potential therapeutic target linking cellular metabolism to tumor progression and immune evasion.

## Introduction

Head and neck squamous cell carcinoma (HNSCCs) is the sixth most common cancer worldwide, posing a significant health challenge (Johnson et al., 2020). The rising incidence of HNSCC is strongly associated with established risk factors, including tobacco use and alcohol consumption. Besides, infection with high-risk strains of human papillomavirus (HPV), particularly HPV-16, is responsible for a growing proportion of cases (Lulic et al., 2024). Despite significant advances in understanding the molecular mechanisms underlying HNSCC, most patients are diagnosed at advanced stages, frequently without detectable pre-malignant lesions, resulting in poor prognosis and high mortality (Antra et al., 2022; Xue et al., 2023). Conventional treatments, encompassing surgery, radiotherapy, and chemotherapy, have shown limited effectiveness in advanced disease and are associated with considerable morbidity (Sun et al., 2023). The limitations of current treatments underscore the urgent need for novel therapeutic strategies and early detection methods to improve patient outcomes.

Recent advances have ushered in the era of immunotherapy, with immune checkpoint inhibitors (ICIs), including pembrolizumab and nivolumab, having been approved for recurrent, metastatic, and unresectable HNSCC (Johnson et al., 2020). However, the overall response rate to ICIs in HNSCC remains suboptimal, with sustained remissions achieved in only a minority of patients (Chen W. et al., 2025). These findings highlight the need to identify key factors and reliable prognostic biomarkers that influence immunotherapeutic efficacy and guide personalized treatment strategies. A growing body of evidence underscores the crucial role of the tumor microenvironment (TME) in HNSCC progression and therapeutic response (Shan et al., 2024). The TME consists of a complex ecosystem, including various immune subsets whose density and functional states significantly impact clinical outcomes (Melssen et al., 2023). Notably, the dichotomy between “cold” tumors, which are immune-excluded, and “hot” tumors, which have immunologically active microenvironments, has become a key determinant of response to immunotherapy across multiple cancer types (Khosravi et al., 2024). In HNSCC, significant heterogeneity in immune cell infiltration has been documented. “Hot” tumors typically exhibit abundant CD8^+^ T cell infiltration, correlating with better prognosis and greater responsiveness to ICIs, while “cold” tumors are characterized by immune cell scarcity and resistance to immunotherapy (Zhou et al., 2022; Zwing et al., 2025; Cao et al., 2022). These findings suggest that the immunological phenotype of the TME may serve as both a prognostic indicator and a therapeutic target. However, the molecular and cellular mechanisms driving this heterogeneity remain incompletely understood, posing challenges to identifying effective biomarkers and treatments.

In recent years, studies have explored immune-related gene signatures and their association with immune cell infiltration patterns to predict prognosis and therapeutic response in HNSCC. For instance, Yang et al. employed a machine learning approach to identify key genes from 1,333 immune-related genes (IRGs) and developed a prognostic model that stratified patients into high- and low-risk groups. The high-risk group demonstrated significantly poorer overall survival (OS) and distinct immune cell infiltration patterns, suggesting that IRGs could effectively differentiate prognostic subgroups and inform clinical decision-making (Yang et al., 2020). Similarly, Chen et al. developed an immune-related gene prognostic index (IRGPI) based on *SFRP4*, *CPXM1*, and *COL5A1*, which independently predicted OS in HNSCC patients. High IRGPI scores were linked to improved OS, increased CD8^+^ T cell and M1 macrophage infiltration, and greater sensitivity to ICI therapy, whereas low IRGPI scores were related to more aggressive phenotypes and an immunosuppressive microenvironment (Chen et al., 2021). Although these signatures demonstrate prognostic value and associations with immune cell infiltration or therapeutic response, further validation is essential to fully realize their potential for clinical application.

In this study, advanced *in silico* techniques were adopted to conduct a comprehensive analysis of immune cell infiltration patterns and their association with immune-related gene expression in HNSCC. Importantly, we sought to elucidate the immunophenotypic differences between immunologically “cold” and “hot” tumors, identify relevant biological pathways, and develop a robust prognostic signature based on IRGs. This research aimed to contribute to the development of more effective, personalized therapeutic strategies for this patient population.

## Materials and methods

### Data collection and processing

Transcriptomic data and corresponding clinical information for HNSCC patients were downloaded from The Cancer Genome Atlas (TCGA) (https://portal.gdc.cancer.gov/) HNSCC cohort (n = 493) and the Gene Expression Omnibus (GEO) (https://www.ncbi.nlm.nih.gov/geo/) datasets GSE41613 (n = 96) and GSE65858 (n = 267). Patients in the TCGA-HNSCC cohort were randomly divided into a training cohort and a test cohort in a 1:1 ratio, and the GSE41613 and GSE65858 datasets were used as external validation cohorts for the constructed signature. The single-cell sequencing raw data were from the GSE103322 dataset (Puram et al., 2017). Immunotherapy cohorts included the IMvigor210 (http://research-pub.gene.com/IMvigor210CoreBiologies/), as well as GSE78220 and GSE91061 datasets. Furthermore, to analyze the mRNA expression and prognostic role of *PYGL*, the TCGA pan-cancer cohort and four GEO datasets (GSE13601, GSE184616, GSE30784, and GSE78060) were utilized. The Human Protein Atlas (https://www.proteinatlas.org/) was referenced to investigate the protein expression of PYGL in HNSCC and normal oral mucosa. A comprehensive summary of all datasets used in this study is provided in Supplementary Table S1.

### Assessment of immune infiltration and identification of immune-related molecular subtypes

To characterize the tumor immune microenvironment and infer the degree of overall immune infiltration, we employed the “ESTIMATE” R package (Yoshihara et al., 2013) to compute immune, stromal, and composite ESTIMATE scores for each sample. The relative abundance of 28 distinct immune cell populations across clusters or risk groups was quantified using single-sample gene set enrichment analysis (ssGSEA) implemented via the “GSVA” R package (Hanzelmann et al., 2013). To enhance robustness and account for potential method-specific biases, we further corroborated these findings through a consensus approach integrating seven established immune deconvolution algorithms: CIBERSORT, CIBERSORT-ABS, EPIC, MCP-counter, quanTIseq, xCell, and TIMER (Sturm et al., 2020).

To explore the biological relevance of IRGs in HNSCC patients, we performed unsupervised consensus clustering using the “ConsensusClusterPlus” R package (Wilkerson and Hayes, 2010). Patient stratification was based on the expression patterns of IRGs, aiming to uncover distinct immune-associated molecular subtypes. The optimal number of clusters (k) was determined by evaluating the cumulative distribution function (CDF) curves across increasing k values and examining the corresponding Δ area plot, which reflects the relative change in the area under the CDF curve. A stable consensus matrix with minimal ambiguity and maximal inter-cluster separation was used to select the optimal cluster number (k).

### Gene set enrichment analysis (GSEA) and functional enrichment analyses

To capture coordinated biological pathway activity beyond individual gene thresholds, we conducted GSEA comparing the C1 and C2 groups using GO gene sets from the Molecular Signatures Database (MSigDB). To identify transcriptional differences between immune-related clusters C1 and C2, we performed differential gene expression analysis using the Wilcoxon rank-sum test (Li et al., 2022). Genes were considered differentially expressed if they met the following criteria: absolute fold change (|FC|) > 1.5 and adjusted *p*-value <0.05. Functional characterization of these DEGs was conducted through Gene Ontology (GO) and Kyoto Encyclopedia of Genes and Genomes (KEGG) enrichment analyses, implemented with the “clusterProfiler” R package (Xu et al., 2024).

### Single-cell RNA sequencing (scRNA-seq) data analysis

The single-cell RNA sequencing dataset GSE103322 was analyzed using the scCancerExplorer platform (http://biocc.hrbmu.edu.cn/scCancerExplorer/), which provides preprocessed and annotated scRNA-seq data derived from the original publication (Huang et al., 2025). The preprocessing steps, including quality control, normalization, dimensionality reduction, clustering, and cell type annotation, were performed by the original study and integrated into the scCancerExplorer database. In the present study, we utilized the processed dataset and annotated cell clusters to evaluate the expression distribution of the six immune-related signature genes across different cell populations. UMAP visualizations and gene expression analyses were directly obtained from the platform. No additional reclustering, normalization, or batch correction was performed.

### Establishment and verification of the IRG prognostic signature

In the TCGA training cohort, we initially performed univariate Cox regression analysis to evaluate the prognostic relevance of IRGs in HNSCC. Subsequently, the least absolute shrinkage and selection operator (LASSO) (using the “glmnet” R package) (Friedman et al., 2010) and stepwise multivariate Cox regression were applied to construct a prognostic signature. The regression coefficients (β) derived from the multivariate Cox proportional hazards model were used to calculate the risk score as a weighted linear combination of gene expression values. The prognostic risk score for each patient was calculated using the formula: Risk Score = ∑ (gene expression × coef). The regression coefficients derived from the TCGA training cohort were fixed and applied unchanged to all validation cohorts for risk score calculation. Patients were classified into high- and low-risk groups using the median risk score within each cohort as the cut-off value. The median cut-off was chosen to minimize overfitting and ensure reproducibility across datasets. Survival differences between the two groups were assessed using Kaplan-Meier survival curves. The prognostic model’s performance was evaluated by constructing the Receiver Operating Characteristic (ROC) curve and calculating the area under the curve (AUC). To evaluate the generalizability and robustness of the model, validation was performed utilizing two internal datasets, namely TCGA-test and TCGA-entire, alongside two external datasets, GSE41613 and GSE65858.

### Association between the IRG signature and clinicopathological characteristics

To explore the clinical relevance of the IRG signature, we examined its association with key clinicopathological variables in patients with HNSCC using data from the TCGA-HNSCC. Visualization of the relationships between risk groups (stratified by the signature) and clinical features was performed using the “ggplot2” and “ggpubr” (https://CRAN.R-project.org/package=ggpubr) R packages. Besides, subgroup survival analyses were conducted across clinically defined categories, with Kaplan-Meier curves used to compare OS between low- and high-risk patients within each subgroup.

### Somatic mutation analysis

We utilized the “maftools” R package (Mayakonda et al., 2018), as well as “ggplot2” and “forestPlot” (https://CRAN.R-project.org/package=forestplot), to analyze somatic variant data from TCGA-HNSCC patients. Tumor mutation burden (TMB) for each patient was calculated, and the relationship between risk score and TMB was subsequently evaluated. The prognostic role of TMB and its combination with risk score was investigated using Kaplan-Meier analysis.

### Development of a prognostic nomogram incorporating the IRG signature

To facilitate clinical translation, we constructed a prognostic nomogram by integrating the risk score derived from the IRGs signature with key clinicopathological variables (e.g., tumor stage, lymphovascular invasion). The nomogram was implemented using the “rms” R package (https://CRAN.R-project.org/package=rms). Its predictive performance for 1-, 3-, and 5-year overall survival was assessed through time-dependent ROC analysis, with the AUC serving as a measure of discriminative accuracy. Model calibration, the agreement between predicted probabilities and observed outcomes, was evaluated using calibration plots. Besides, the concordance index (C-index) was calculated to quantify the overall predictive concordance of the nomogram. Finally, patients were stratified into distinct risk groups based on nomogram-derived scores, and Kaplan-Meier survival analysis with log-rank testing was performed to validate the model’s ability to discriminate survival outcomes.

### Weighted gene co-expression network analysis (WGCNA)

To uncover gene networks associated with the IRG signature, we performed WGCNA on the TCGA-HNSCC transcriptomic data using the “WGCNA” R package (Langfelder and Horvath, 2008). A soft-thresholding power of 12 was selected to approximate a scale-free topology (scale-free topology fit index >0.90). Hierarchical clustering grouped co-expressed genes into 12 distinct modules, each assigned a unique color label. The module exhibiting the strongest correlation with the IRG signature, based on module eigengene–trait association, was prioritized for downstream functional characterization. Functional annotation and pathway enrichment analyses of this module were performed using the “clusterProfiler”, “org.Hs.eg.db”, and “enrichplot” (https://bioconductor.org/packages/enrichplot/) R packages, enabling systematic annotation of biological processes, cellular components, molecular functions, and KEGG pathways. Moreover, GSEA was employed to evaluate the differential pathway enrichment between groups classified as high-risk and low-risk. The analyses were performed utilizing the “clusterProfiler” package in R, with pathways deemed significantly enriched if the adjusted *p*-value was less than 0.05.

### Immunotherapy prediction and drug sensitivity analysis

Differential expression of key immune checkpoint molecules (e.g., PD-1, CTLA-4, LAG3, TIGIT) between the low- and high-risk groups was systematically evaluated to assess potential implications for immunotherapy response. To explore the relationship between the prognostic signature and response to immunotherapy, we collected two GEO immunotherapy cohorts with the IMvigor210 cohort. In addition, drug sensitivity analysis was performed using the NCI-60 cancer cell line panel, which represents a diverse spectrum of human cancer cell lines and provides comprehensive response data for 792 chemotherapeutic and targeted agents (Ren et al., 2025). Specifically, we investigated the association between the calculated risk scores and the half-maximal inhibitory concentration (IC_50_) values of these agents, thereby assessing the potential predictive value of the signature for therapeutic response. All statistical analyses and graphical visualizations were conducted using the “ggplot2” package in R.

### Cell culture

The HNSCC cell lines SCC-9 (Cat.No.CL-0571) and SAS (Cat.No.CL-0849) were purchased from the Procell (Wuhan, China). Cell identity was confirmed by short tandem repeat (STR) profiling, and all cultures were routinely tested and confirmed to be mycoplasma-free prior to experimental use. SCC-9 cells were maintained in DMEM/F12 medium (Gibco, United States), while SAS cells were cultured in DMEM (Gibco, United States). Both cell lines were grown in media supplemented with 10% fetal bovine serum (FBS; Gibco, United States) and 1% penicillin–streptomycin (Solarbio, China, Cat. No. P1400). Cells were incubated at 37 °C in a humidified atmosphere containing 5% CO_2_.

### PYGL silencing

To silence PYGL expression, three custom-designed small interfering RNAs (siRNAs) were synthesized by GenePharma (Suzhou, China). A non-targeting scrambled siRNA (si-NC) was used as a negative control, while two distinct siRNAs targeting PYGL (si-PYGL-1 and si-PYGL-2) were employed for knockdown experiments. The sequences were as follows: si-NC sense: 5′-UUC​UCC​GAA​CGU​GUC​ACG​UTT-3′, si-NC antisense: 5′-ACG​UGA​CAC​GUU​CGG​AGA​ATT-3′, si-PGYL-1 sense: 5′-GCA​CGC​AGC​AGC​ACU​ACU​ATT-3′, si-PGYL-1 antisense: 5′-UAG​UAG​UGC​UGC​UGC​GUG​CTT-3′, si-PGYL-2 sense: 5′-CCU​GGA​GAC​GGA​GUA​CAA​ATT-3′, si-PGYL-2 antisense: 5′-UUU​GUA​CUC​CGU​CUC​CAG​GTT-3′. SCC-9 and SAS cells were seeded at 2 × 10^5^ cells per well in six-well plates and allowed to adhere overnight. Upon reaching 70%–80% confluence, cells were transfected with either si-NC or one of the two PYGL-targeting siRNAs using Lipofectamine 3000 (Thermo Fisher Scientific, Cat. No. L3000015, United States), following the manufacturer’s recommended protocol.

### Real-time quantitative polymerase chain reaction (RT-qPCR) assay

To investigate the expression of PYGL knockdown, we assessed the mRNA levels of PYGL in SCC-9 and SAS cells. Total RNA was extracted using the SevenFast® Total RNA Extraction Kit for Cells (Cat. No. SM130, SevenBio, China) following the manufacturer’s protocol. RNA concentration and purity were measured with a NanoDrop One spectrophotometer (Thermo Fisher Scientific, United States). First-strand cDNA was synthesized from 1 μg of total RNA using the TransScript® Uni All-in-One First-Strand cDNA Synthesis SuperMix for qPCR (One-Step gDNA Removal; Cat. No. AU341, TransGen Biotech, China), as per the manufacturer’s instructions.

RT-qPCR was performed on a QuantStudio™ 6 Real-Time PCR System (Thermo Fisher Scientific, United States) using MicroAmp Fast 0.2 mL 96-well Reaction Plates (Thermo Fisher Scientific, United States) and PowerUp™ SYBR™ Green Master Mix (Cat. No. A25742, Thermo Fisher Scientific, United States). GAPDH was used as an internal reference gene, and relative PYGL mRNA expression was calculated using the 2^^(−ΔΔCt)^ method. All primers were synthesized by Sangon Biotech (Shanghai, China) and the sequences were as follows: GAPDH forward: 5′-TTG​CCA​TCA​ATG​ACC​CCT​TCA-3′, GAPDH reverse: 5′-CGC​CCC​ACT​TGA​TTT​TGG​A-3’. PYGL forward: 5′- CAG​CCT​ATG​GAT​ACG​GCA​TTC-3′, PYGL reverse: 5′- CGG​TGT​TGG​TGT​GTT​CTA​CTT​T-3’.

### Assessment of cell proliferation following PYGL knockdown

To evaluate the functional impact of PYGL silencing on cellular proliferation, two complementary assays were employed. First, cell viability was assessed using the Cell Counting Kit-8 (CCK-8; Biosharp, Cat.No.BS350A, China). SCC-9 and SAS cells transfected with non-targeting control siRNA (si-NC) or two distinct PYGL-targeting siRNAs (si-PYGL-1 and si-PYGL-2) were seeded into 96-well plates at a density of 2,000 cells per well. At designated time points (0, 24, 48, and 72 h), 10 μL of CCK-8 reagent was added to each well, followed by 2 h incubation at 37 °C in a humidified 5% CO_2_ atmosphere. Absorbance at 450 nm was measured using a using a Varioskan LUX microplate reader (Thermo Fisher Scientific, United States). Each experiment was performed with five biological replicates to ensure reproducibility.

In parallel, colony formation assay was also performed. Cells were seeded into 12-well plates at 2,000 cells per well and cultured for 7 days under standard conditions. Colonies were then fixed with 4% paraformaldehyde (Solarbio, Cat. No. P1110, China), stained with 0.1% crystal violet (Solarbio, Cat. No. G1062, China), and imaged using a digital camera. The number of colonies were counted manually using ImageJ software (National Institutes of Health, United States). All experiments were performed in triplicate.

### Cell migration assay

The migratory capacity of SCC-9 and SAS cells following PYGL knockdown was evaluated using an *in vitro* wound healing assay. Briefly, cells transfected with control siRNA (si-NC) or two independent PYGL-targeting siRNAs (si-PYGL-1 and si-PYGL-2) were resuspended in complete culture medium at a density of 5 × 10^5^ cells/mL. A total of 70 μL of cell suspension was seeded into each chambel of the Culture-Insert (Cat. No. 80209, ibidi, Germany). After 24 h of incubation to allow cell adhesion and monolayer formation, the insert was carefully removed to create a reproducible cell-free gap. Images of the wound area were captured at 0, 6, and 9 h post-scratch under a phase-contrast inverted microscope at ×10 magnification. Wound closure was quantified using ImageJ software (National Institutes of Health, United States) by measuring the relative change in gap area. The migration rate was calculated as: Migration rate (%) = [(A_0_ − A_t_)/A_0_] × 100, where A_0_ is the initial wound area and A_t_ is the area at the indicated time point. All experiments were performed independently in triplicate. The experimental procedure was carried out as previously described (Pang et al., 2025).

### Transcriptome sequencing following PYGL gene knockdown

To explore the molecular mechanisms of PYGL knockdown in HNSCC, we performed RNA sequencing on SAS cells transfected with si-NC or si-PYGL. Each group included five biological replicates. RNA-seq library preparation and sequencing were performed by Personalbio (http://www.personalbio.cn/) (China). DEGs were defined as those with an adjusted *p*-value <0.05 and an absolute fold change >1.5. GO analysis was performed using the “clusterProfiler”, “org.Hs.eg.db”, and “enrichplot” R packages. GSEA was performed to identify significantly enriched hallmark pathways between the two groups, with pathways considered significant at an adjusted *p*-value <0.05.

### Statistical analysis

All statistical analyses were conducted using R software (v4.2.2). For comparisons between two independent groups, normally distributed data were analyzed using the two-tailed Student’s t-test, and non-normally distributed data were assessed with the Wilcoxon rank-sum test. Associations between continuous variables were evaluated using Pearson’s rank correlation coefficient. Statistical significance was denoted as follows: *p* < 0.05 (*), *p* < 0.01 (**), *p* < 0.001 (***), and *p* < 0.0001 (****); non-significant results were labeled “ns”.

## Results

### Identification of immune infiltration-related consensus clusters

**FIGURE 1:**
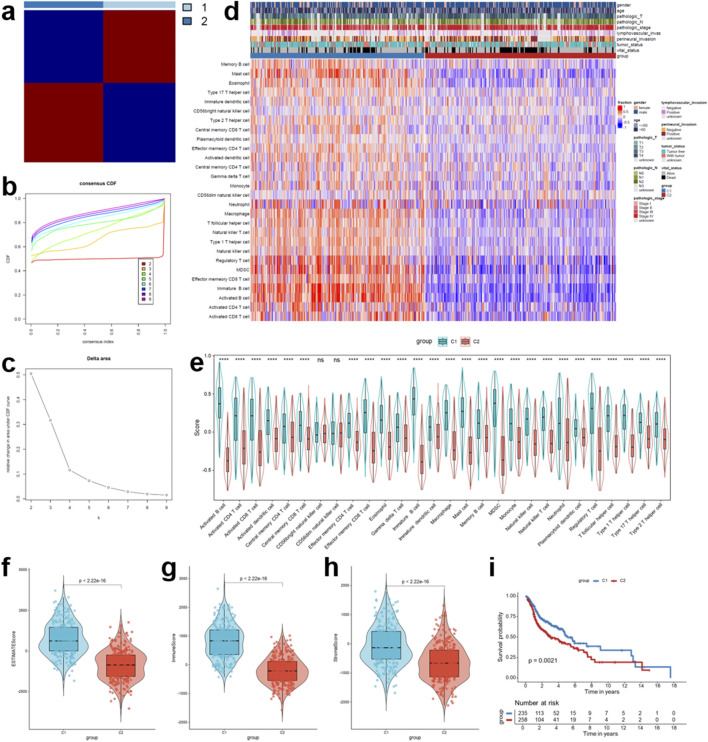
Identification of immune infiltration-related consensus clusters in TCGA-HNSCC cohort. **(a)** Consensus clustering heatmap of all samples at k = 2. **(b)** CDF curves of consensus matrix for each k value. **(c)** The relative change in the area under the CDF curves with distinct k values. **(d)** Heatmap for the infiltration abundance of 28 immune cell populations assessed by ssGSEA for the two clusters (C1 and C2), along with several important clinicopathological characteristics of HNSCC patients. **(e)** Comparison of 28 immune cell populations infiltration between the two clusters. **(f–h)** The distribution of the immune score calculated by the ESTIMATE algorithm, including the ESTIMATE, Immune, and Stromal scores, between the two clusters. **(i)** Kaplan-Meier curves of overall survival (OS) between the two clusters in TCGA-HNSCC cohort (log-rank test: *p* = 0.0021). ****p < 0.0001, ns: no significance.

Based on the infiltration levels of 28 immune cell types quantified by ssGSEA, we obtained several prognostic immune cell types in TCGA-HNSCC cohort by conducting univariate Cox regression, including “Type 17 T helper cell”, “Eosinophil”, “Activated B cell”, “Mast cell”, “Immature B cell”, “Central memory CD8 T cell”, and “CD56bright natural killer cell” (Supplementary Table S2). Subsequently, consensus clustering analysis was performed using these immune cells, with the number of clusters (k) ranging from 2 to 9. As shown in the consensus heatmap, at k = 2, the samples could be robustly divided into two distinct and stable clusters, characterized by high intra-cluster similarity and low inter-cluster correlation (Figure 1a). The CDF curves further supported the robustness of clustering, exhibiting progressively smoother distributions with increasing k (Figure 1b). In addition, the relative change in the area under the CDF curve exhibited a sharp increase from k = 2 to k = 3, whereas subsequent increases were minimal, indicating that k = 2 represented the most stable clustering solution (Figure 1c).

Subsequent analysis revealed clear differences in immune infiltration patterns between the two clusters. Cluster 1 (C1) exhibited markedly higher infiltration of multiple immune cell populations compared to Cluster 2 (C2) (Figures 1d,e). To further characterize the TME of HNSCC samples, the ESTIMATE algorithm was applied, which showed that ESTIMATE scores, Immune scores, and Stromal scores were all significantly higher in C1 than in C2 (all *p* < 0.05) (Figures 1f–h). Importantly, survival analysis indicated that patients in C2 exhibited significantly worse overall outcomes than those in C1 (Figure 1i). Taken together, these findings suggest that immune infiltration-based consensus clustering can stratify HNSCC patients into two biologically and clinically distinct subgroups, with C1 representing an “immune-hot” phenotype associated with a favorable prognosis, and C2 corresponding to an “immune-cold” phenotype linked to poor clinical outcomes.

### Functional enrichment analysis of immune infiltration-related clusters in HNSCC

**FIGURE 2:**
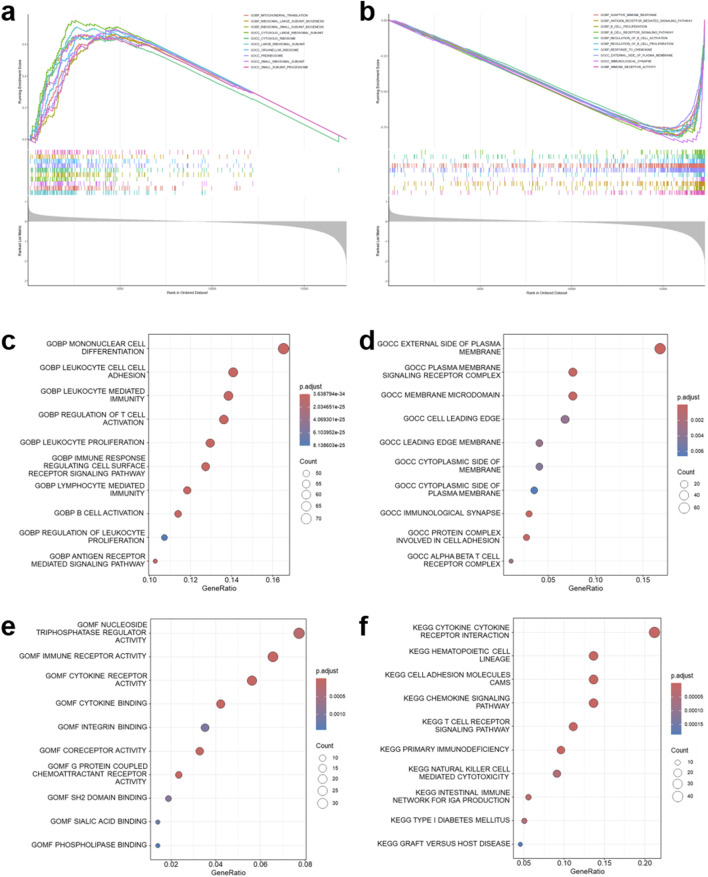
Functional enrichment analyses between immune infiltration-related consensus clusters in TCGA-HNSCC cohort. **(a,b)** Gene set enrichment analysis (GSEA) plots comparing the C2 cluster versus the C1 cluster, highlighting the key biological pathways that are positively and negatively enriched in the C2 cluster, respectively. **(c–e)** Gene Ontology (GO) enrichment analysis of prognostic differentially expressed genes (DEGs) between the C2 cluster versus the C1 cluster. **(f)** Kyoto Encyclopedia of Genes and Genomes (KEGG) pathway enrichment analysis of prognostic DEGs.

To further investigate the biological characteristics of the immune infiltration-related clusters (C1 and C2) in HNSCC, GSEA was conducted. Compared with C1, cluster C2 was positively enriched in ribosome-related processes, including mitochondrial translation, ribosomal subunit biogenesis, and cytosolic as well as organellar ribosome components (Figure 2a). In contrast, hallmark sets negatively enriched in C2 were mainly immune-related, such as adaptive immune response, antigen receptor-mediated signaling, B cell receptor signaling, regulation of B cell activation and proliferation, response to chemokine, and immunological synapse (Figure 2b).

Furthermore, differential expression analysis (fold change >1.5, *p* < 0.05) between C1 and C2 clusters identified 1225 genes (Supplementary Table S3), among which 491 genes were further confirmed to have prognostic value by univariate Cox regression analysis (Supplementary Table S4). Functional enrichment analysis of these genes demonstrated a predominant involvement in immune-related processes. GO analysis revealed significant enrichment in immune activation, leukocyte cell-cell adhesion, and cytokine regulation (Figures 2c–e). KEGG analysis further highlighted canonical immune-related pathways, including cytokine-cytokine receptor interaction, cell adhesion molecules, chemokine signaling, and T cell receptor signaling (Figure 2f). Overall, these findings confirm the biological dichotomy between the two clusters, suggesting that C2 represents a translationally active yet immunologically altered phenotype.

### Construction of a prognostic IRG signature for HNSCC patients

**FIGURE 3:**
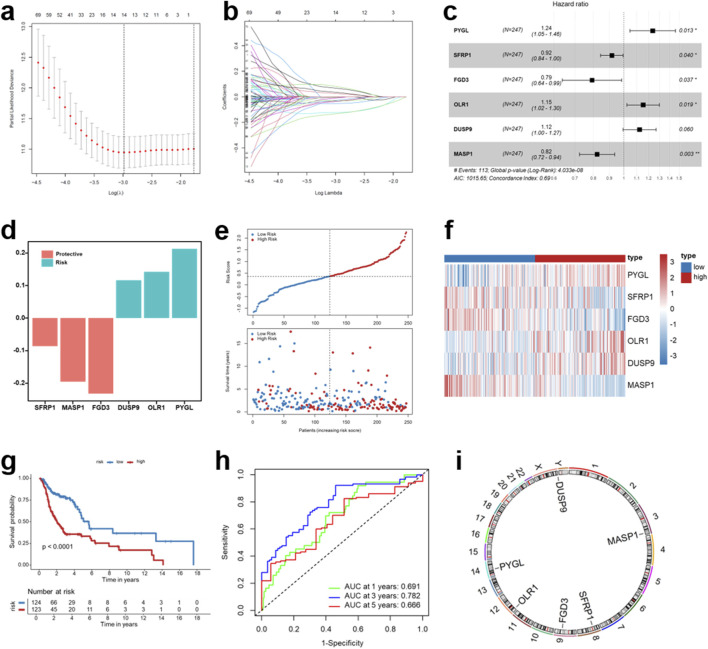
Construction of a prognostic IRG signature for HNSCC patients. **(a,b)** Construction of a prognostic immune-related signature by the LASSO Cox regression analysis in the TCGA-HNSCC training cohort. **(c)** Multivariate Cox regression analysis for the six genes in the identified signature. **(d)** The bar plot illustrates the role of selected genes in the signature associated with prognosis in HNSCC patients. **(e)** Distribution of risk scores and survival status of HNSCC patients. **(f)** Expression profiles of the prognostic signature between the low- and high-risk groups. **(g)** Kaplan-Meier survival analysis for the overall survival (OS) of HNSCC patients between the two risk groups. **(h)** Receiver operating characteristic (ROC) curve analysis for 1-year, 3-year, and 5-year OS of HNSCC patients. **(i)** Chromosomal locations of the six immune-related genes. *p < 0.05, **p < 0.01.

Next, we performed univariate Cox regression and LASSO Cox regression using DEGs in the TCGA-HNSCC training cohort, which yielded a total of 14 candidate genes: *PYGL, NQO1, SFRP1, INPP5D, TMC8, FGD3, OLR1, PRSS12, DUSP9, MASP1, TMEM150C, CR2, CTSG,* and *HDC* (Figures 3a,b). Subsequent stepwise multivariate Cox regression analysis yielded a prognostic signature comprising six genes (*PYGL*, *SFRP1*, *FGD3*, *OLR1*, *DUSP9*, and *MASP1*) (Figure 3c). The C-index of the model reached 0.69, indicating moderate predictive performance. Among these genes, *DUSP9*, *OLR1*, and *PYGL* were identified as risk-associated genes, whereas SFRP1, MASP1, and FGD3 functioned as protective factors (Figure 3d). Based on the six-gene signature, we calculated a risk score for each patient: Risk score = 0.212527597 × PYGL +0.142335469 × OLR1 + 0.116373923 × DUSP9 + (−0.086414273) × SFRP1 + (−0.194882676) × MASP1 + (−0.232095756) × FGD3. The distribution of risk scores, survival status, and corresponding expression patterns of the six genes is shown in Figures 3e,f. Patients in the high-risk group exhibited significantly higher mortality compared with those in the low-risk group. Kaplan-Meier survival analysis further confirmed that the high-risk group exhibited markedly poorer outcomes (*p* < 0.001) (Figure 3g). Time-dependent ROC analysis demonstrated satisfactory predictive accuracy of the signature, with AUC values of 0.691, 0.782, and 0.666 for 1-, 3-, and 5-year survival, respectively (Figure 3h). The genomic distribution of the six genes is presented in Figure 3i, highlighting their chromosomal localization. Collectively, these results suggest that the six-gene immune-related signature provides robust prognostic stratification in HNSCC and may serve as a valuable tool for risk assessment and clinical decision-making.

### ScRNA-seq analysis of IRGs in HNSCC samples

**FIGURE 4:**
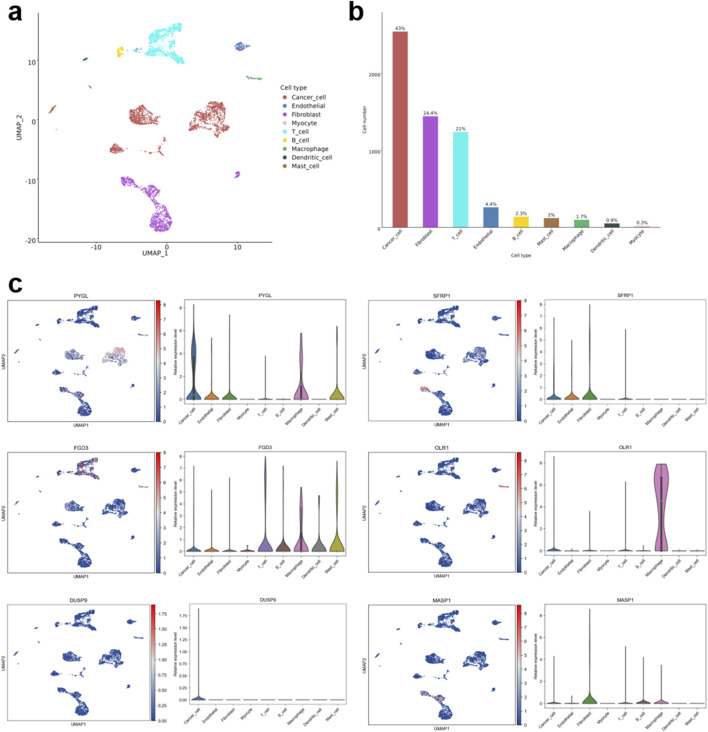
Single-cell RNA sequencing (scRNA-seq) analysis for HNSCC. **(a)** Uniform manifold approximation and projection (UMAP) plot of scRNA-seq data from dataset GSE103322 using the scCancerExplorer online database. **(b)** Quantitative analysis of distinct cell types. **(c)** Relative expression level of six immune-related signature genes across distinct cell types.

By fully leveraging the scCancerExplorer database, we systematically analyzed the single-cell RNA sequencing dataset GSE103322 of HNSCC. Uniform manifold approximation and projection (UMAP)-based dimensionality reduction identified distinct cellular subpopulations within the TME, including cancer cells, endothelial cells, fibroblasts, myocytes, T cells, B cells, macrophages, dendritic cells, and mast cells (Figure 4a). Quantitative analysis revealed that cancer cells constituted the largest fraction (43%), followed by fibroblasts (24.4%) and T cells (21%), whereas endothelial cells (4.8%), B cells (2.3%), myocytes (2%), mast cells (2%), macrophages (1.7%), dendritic cells (0.3%), and myocytes (0.3%) represented relatively minor subsets (Figure 4b).

Expression profiling of the six immune-related prognostic signature genes demonstrated distinct cell type-specific patterns (Figure 4c). *PYGL* was predominantly expressed in cancer cells, with additional elevation in macrophages and mast cells. *SFRP1* was strongly enriched in fibroblasts, and to a lesser extent, in endothelial cells. *FGD3* exhibited its highest and broadest expression in mast cells, with appreciable levels in B cells and dendritic cells, and more restricted expression in small subsets of T cells and macrophages. *OLR1* was strongly enriched in macrophages, consistent with tumor-associated macrophages (TAMs), but also showed moderate expression in cancer cells. *DUSP9* showed cancer cells-specific expression, whereas *MASP1* exhibited low but relatively broad expression, with modest enrichment in fibroblasts and macrophages. Overall, these findings highlight the marked cellular heterogeneity of HNSCC and underscore the potential functional and prognostic roles of IRGs in shaping the tumor immune microenvironment.

### Evaluation of the IRG signature in independent HNSCC cohorts

**FIGURE 5:**
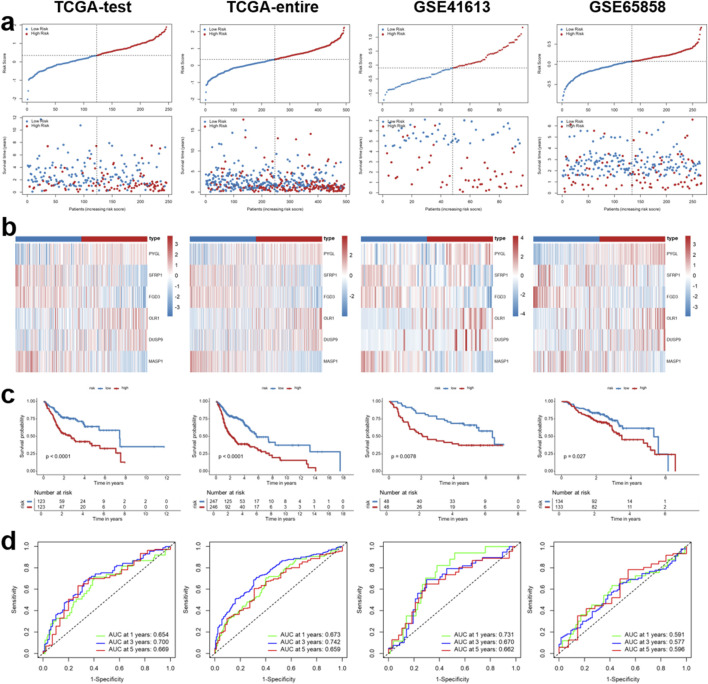
Evaluation of the IRG signature in independent HNSCC cohorts. **(a)** Distribution of risk scores and survival status of HNSCC patients across multiple validation cohorts, including TCGA-test, TCGA-entire, GSE41613, and GSE65858 datasets. **(b)** Expression profiles of the six immune-related signature genes in the low- and high-risk groups across the validation cohorts. **(c)** Kaplan-Meier survival analysis of the overall survival (OS) for HNSCC patients in the low- and high-risk groups across the validation cohorts. **(d)** Receiver operating characteristic (ROC) curve analyses for predicting 1-, 3-, and 5-year overall survival (OS) across the validation cohorts.

To evaluate the robustness and generalizability of the immune-related prognostic signature, we performed internal and external validation in four HNSCC cohorts, including TCGA-test, TCGA-entire, GSE41613, and GSE65858. The distribution of risk scores and survival status consistently showed that patients in the high-risk group exhibited a greater proportion of deaths compared with those in the low-risk group across all datasets (Figure 5a). Correspondingly, heatmaps of gene expression patterns revealed distinct expression differences between the two groups, highlighting the discriminative capacity of the signature (Figure 5b). Kaplan-Meier survival analyses further demonstrated that high-risk patients exhibited significantly poorer OS than low-risk patients in each cohort (all *p* < 0.05, Figure 5c). Importantly, time-dependent ROC curve analyses confirmed the predictive accuracy of the model, with the AUC values supporting stable prognostic performance at different time points across cohorts (Figure 5d). Collectively, these findings validate that the immune-related prognostic signature is a robust and reliable predictor of survival outcomes in HNSCC patients, with consistent performance across multiple independent datasets.

### Association between the IRG signature and clinicopathological features in HNSCC patients

To explore the clinical relevance of the immune-related prognostic signature, we examined the association between risk scores and various clinicopathological features in the TCGA-HNSCC cohort. The clinical parameters assessed included age, lymphovascular invasion, perineural invasion, tumor status, clinical TNM stage, overall clinical stage, pathological T/N/M stage, and overall pathological stage (Supplementary Figure Sa-1). Patients with higher risk scores were significantly more likely to present with perineural invasion (Supplementary Figure S1c), positive tumor status (Supplementary Figure S1d), advanced clinical T stage (Supplementary Figure S1e), higher overall clinical stage (Supplementary Figure S1h), advanced pathological T stage (Supplementary Figure S1i), pathological N stage (Supplementary Figure S1j), and higher overall pathological stage (Supplementary Figure S1l) (all *p* < 0.05). No significant associations were observed with other clinicopathological factors. These findings suggest that the risk score derived from the immune-related prognostic signature is closely linked to aggressive clinicopathological characteristics, reflecting its potential role in capturing the malignant progression of HNSCC patients.

### Prognostic value of the IRG signature across clinical subgroups

To determine whether the prognostic power of the immune-related gene signature was maintained across different clinical contexts, we conducted Kaplan-Meier subgroup survival analyses in the TCGA-HNSCC cohort (Supplementary Figure S2). Stratification by age, sex, tumor status, clinical TNM stage, overall clinical stage, pathological TNM stage, and overall pathological stage consistently demonstrated that patients in the high-risk group exhibited significantly poorer OS compared with those in the low-risk group across all subgroups. These findings highlight the robust and independent prognostic value of the immune-related gene signature, confirming its utility for risk stratification in HNSCC patients irrespective of clinicopathological background.

### Analysis for genomic alterations and TMB associated with the IRG signature

**FIGURE 6:**
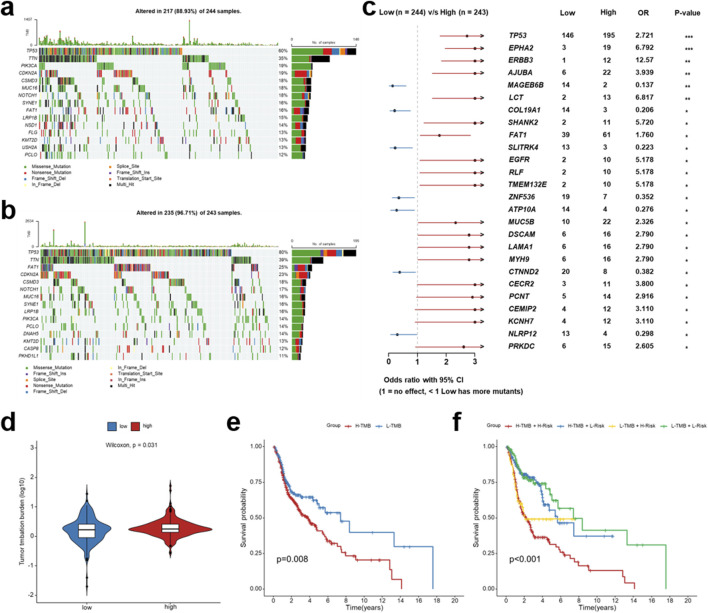
Genomic alterations and tumor mutation burden (TMB) analysis associated with an immune-related signature in HNSCC patients. **(a,b)** Mutational landscape of the top mutated genes in the low-risk group **(a)** and high-risk group **(b)** based on the IRG signature from TCGA-HNSCC patients. Mutation types are color-coded, with mutation frequencies displayed on the right. **(c)** Forest plot comparing significantly mutated genes between the low- and high-risk groups, showing odds ratio (OR) and 95% confidence interval (95%CI). **(d)** Comparison of TMB levels between the low- and high-risk groups. **(e)** Kaplan-Meier survival curves for HNSCC patients stratified by TMB status. **(f)** Kaplan-Meier survival analysis of four subgroups defined by both risk score and TMB status. *p < 0.05, **p < 0.01, ***p < 0.001.

We next investigated the mutational landscape of TCGA-HNSCC patients stratified by the immune-related signature. Both the low- and high-risk groups exhibited frequent alterations in common driver genes such as *TP53*, *TTN*, *FAT1*, and *CDKN2A*, although their mutational frequencies varied substantially (Figures 6a,b). Comparative analyses revealed several genes with significantly different mutation rates between the two groups. As shown in the forest plot (Figure 6c), mutations in *TP53*, *EPHA2*, *ERBB3*, *FAT1*, and *EGFR* were more prevalent in high-risk patients, whereas alterations in *COL19A1*, *ZNF536*, and *NLRP12* were enriched in the low-risk group. These results suggest that prognostic stratification based on the immune-related signature reflects distinct genomic backgrounds.

We further examined the relationship between the prognostic signature and TMB. High-risk patients displayed significantly higher TMB (*p* = 0.031, Figure 6d) and worse overall survival (*p* = 0.008, Figure 6e) compared with the low-risk group. Notably, integrating TMB status with the risk score refined prognostic stratification: patients with both low-risk score and low TMB achieved the most favorable outcomes, whereas those with high-risk score and high TMB exhibited the worst survival (*p* < 0.001, Figure 6f). The above findings indicate that the immune-related signature not only mirrors the underlying genomic alterations and mutational burden in HNSCC but also enhances prognostic precision by integrating molecular and genomic features.

### Nomogram integrating the IRG signature for individualized survival prediction in HNSCC

**FIGURE 7:**
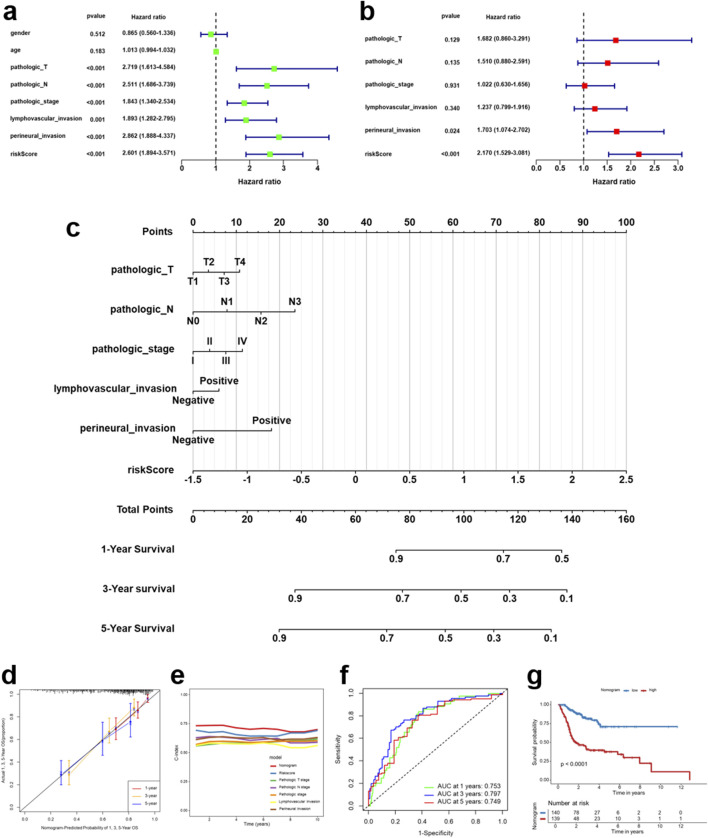
A nomogram incorporating the IRG signature to predict prognosis in HNSCC patients. **(a,b)** Univariate **(a)** and multivariate **(b)** Cox regression analyses assessing the prognostic significance of the risk score and key clinicopathologic features in TCGA-HNSCC patients. **(c)** Nomogram integrating significant prognostic factors for predicting 1-year, 3-year, and 5-year overall survival (OS) in HNSCC patients. **(d)** Calibration curves showing the agreement between predicted and observed survival probabilities at 1, 3 and 5 years. **(e)** Time-dependent concordance index (C-index) for the nomogram and individual prognostic factors. **(f)** Receiver operating characteristic (ROC) curves assessing the predictive performance of the nomogram at 1, 3, and 5 years. **(g)** Kaplan-Meier survival analysis stratifying patients into low- and high-risk groups based on the nomogram score, with survival differences evaluated using the log-rank test.

Univariate and multivariate Cox regression analyses demonstrated that the risk score derived from the immune-related gene signature served as an independent prognostic factor for OS in TCGA-HNSCC patients, even after adjusting for conventional clinicopathologic variables (Figures 7a,b). Based on these significant predictors, a prognostic nomogram was generated to estimate 1-, 3-, and 5-year OS rates (Figure 7c). The calibration curves revealed excellent agreement between the predicted and observed outcomes, underscoring the accuracy of the model (Figure 7d). Time-dependent C-index analyses showed that the nomogram consistently achieved higher predictive performance than individual clinical parameters alone (Figure 7e). Similarly, ROC curve analyses confirmed the robustness of the model, with AUC values of 0.753, 0.797, and 0.749 for survival at 1, 3, and 5 years, respectively (Figure 7f). Furthermore, patients stratified by the nomogram into high- and low-risk groups showed significantly different overall survival, with markedly poorer outcomes in the high-risk group (*p* < 0.001, Figure 7g). These results demonstrate that the nomogram, which integrates the immune-related gene signature with clinical variables, provides a reliable and clinically applicable tool for personalized prognostic assessment in HNSCC.

### WGCNA and GSEA of the IRG signature

To further elucidate the biological mechanisms underlying the prognostic signature, we performed WGCNA based on TCGA-HNSCC transcriptomic data. Scale-free topology was confirmed, indicating the reliability of the constructed co-expression network (Supplementary Figure S3a). Hierarchical clustering identified 12 distinct co-expression modules, each represented by a unique color. Among these, the yellow module showed the strongest correlation with the risk groups (correlation = −0.37, *p* = 4e-17), suggesting its close association with prognosis (Supplementary Figures S3b, S3c). Moreover, a strong positive association between gene significance and module membership was observed within this module (correlation = 0.47, *p* = 1.3e-80), underscoring its biological importance (Supplementary Figure S3d). Functional enrichment analyses demonstrated that genes in the yellow module were predominantly involved in immune-related processes. GO analysis revealed significant enrichment in terms such as leukocyte-mediated immunity, regulation of immune effector process, and regulation of T cell activation (Supplementary Figure S3e). KEGG pathway analysis yielded consistent results, highlighting significant enrichment in cytokine-cytokine receptor interactions, chemokine signaling, and cell adhesion molecules (Supplementary Figure S3f).

To gain a more profound understanding of the biological processes that underlie the prognostic differences identified between high- and low-risk cohorts defined by the IRG signature, GSEA was performed. GSEA revealed a distinct molecular profile in the high-risk group, characterized by activation of ribosome biogenesis and protein translation machinery alongside suppression of immune receptor-mediated signaling pathways (Supplementary Figures S3g–i). Ribosome-related processes, including ribosomal small subunit biogenesis, cytosolic large ribosomal subunit, structural constituent of ribosome, and pre-ribosome, were significantly positively enriched, indicating enhanced translational capacity. In contrast, multiple immune-related pathways, such as adaptive immune response, alpha-beta T cell activation, antigen receptor-mediated signaling, B cell receptor signaling, and immune receptor activity, were negatively enriched, reflecting impaired activation and coordination of key immune cell subsets. This combination of upregulated protein synthesis and attenuated immune signaling suggests a tumor-promoting state in high-risk patients, potentially facilitating immune evasion and rapid tumor progression.

### Immune infiltration patterns associated with the IRG signature

**FIGURE 8:**
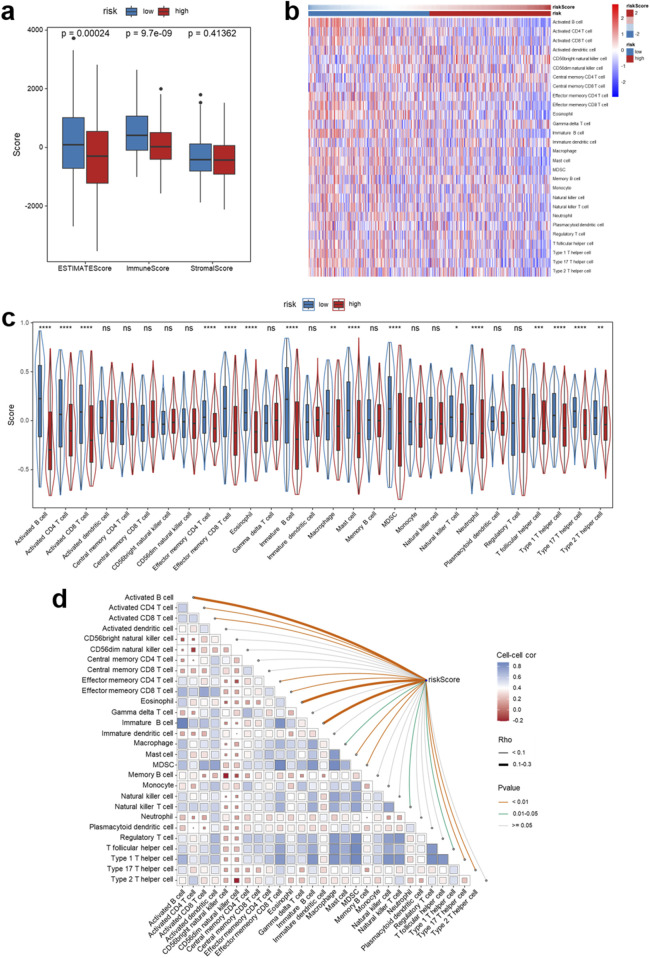
Identification of immune infiltration characteristics of the signature in the TCGA-HNSCC cohort. **(a)** Comparison of ESTIMATE, Immune, and Stromal scores between low- and high-risk groups defined by the signature. **(b)** Heatmap displaying the distribution of 28 immune cell subpopulations across low- and high-risk groups. **(c)** Violin plots showing differences in the relative abundance of various immune cell subtypes between the low- and high-risk groups. **(d)** Correlation matrix illustrating the relationships between immune cell abundance and the risk score. *p < 0.05, **p < 0.01, ***p < 0.001, ****p < 0.0001, ns: not significant.

Immune infiltration analysis in the TCGA-HNSCC cohort using the ESTIMATE algorithm revealed distinct differences between risk groups defined by the IRG signature. High-risk patients displayed markedly reduced ESTIMATE score (*p* = 0.00024) and immune score (*p* = 9.7e−09), while the stromal score remained comparable (*p* = 0.41362) (Figure 8a). Figure 8b presents a heatmap showing the distribution of 28 immune cell subpopulations across low- and high-risk groups. Besides, a violin plot (Figure 8c) was generated to compare the risk scores between these groups. Correlation matrix analysis further confirmed these results, indicating a negative relationship between risk score and effector immune cell subsets (Figure 8d). To validate alterations in the immune landscape, we employed various algorithms for immune cell deconvolution, such as CIBERSORT, TIMER, xCell, EPIC, MCP-counter, and quanTIseq. Taken together, these findings indicate that patients classified as high-risk demonstrate decreased infiltration of anti-tumor immune cells, contributing to an immunosuppressive tumor microenvironment (Supplementary Figure S4). Collectively, these results demonstrate a strong association between the IRG signature and specific immune infiltration patterns in the tumor microenvironment of HNSCC.

## Implications of signature for immune checkpoint blockade (ICB) treatment

**FIGURE 9:**
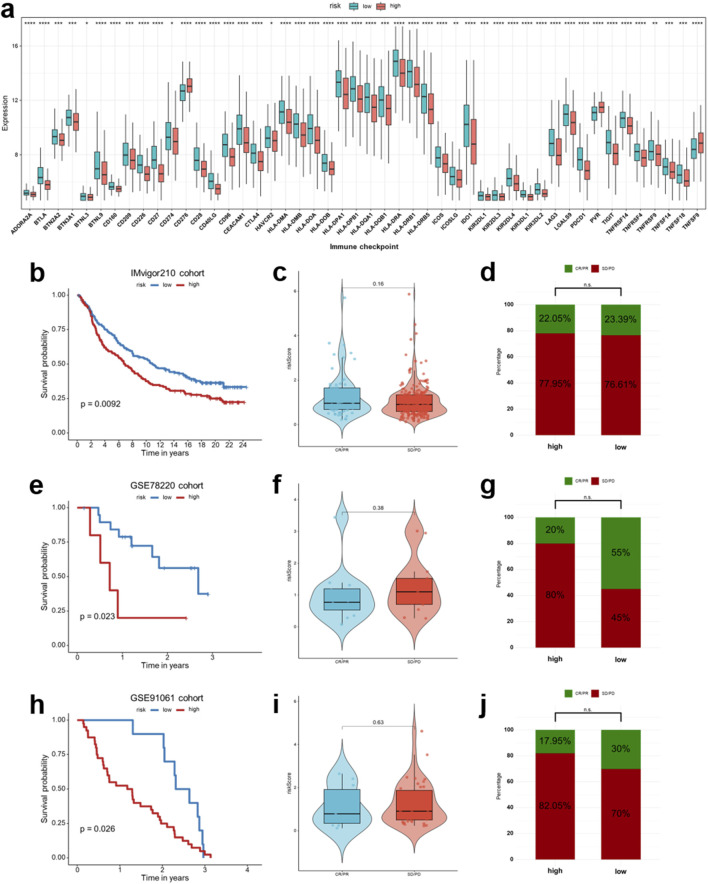
Association of the IRG signature with immune checkpoint expression and immunotherapy response. **(a)** Boxplots comparing the expression of immune checkpoint molecules between low- and high-risk groups in the TCGA-HNSCC cohort. **(b,e,h)** Survival analysis of the IMvigor210, GSE78220 and GSE91061 immunotherapy cohort, respectively. **(c,f,i)** Distribution of risk scores between complete/partial response (CR/PR) and stable/progressive disease (SD/PD) groups following immune checkpoint blockade (ICB) therapy in the above cohorts. **(d,g,j)** Proportion of CR/PR and SD/PD cases in the low- and high-risk groups for the aforementioned cohorts. *p < 0.05, **p < 0.01, ****p < 0.0001, ns: not significant.

Given the clinical significance of immune checkpoints and immunotherapy in HNSCC, we first assessed whether the risk score was associated with differential immune checkpoint expression. In the TCGA-HNSCC cohort, low-risk patients exhibited significantly higher expression of multiple immune checkpoint molecules, including PDCD1 (PD-1), CD274 (PD-L1), CTLA-4, and several other inhibitory receptors (Figure 9a). This pattern reflects an immune-inflamed tumor microenvironment characterized by enhanced immune regulatory activity, which may indicate a higher likelihood of responsiveness to immune checkpoint blockade therapy. We next evaluated the predictive value of the risk score in three independent immunotherapy cohorts (IMvigor210, GSE78220, and GSE91061). In all three datasets, low-risk patients consistently demonstrated superior OS following ICB therapy compared to those in the high-risk group (IMvigor210, *p* = 0.0092; GSE78220, *p* = 0.023; GSE91061, *p* = 0.026; Figures 9b,e,h). We further investigated the correlation between the risk score and the status of clinical response, differentiating between patients with clinical benefit (complete/partial response, CR/PR) and those with non-response (stable/progressive disease, SD/PD). Although the distribution of risk scores across different response categories did not reach statistical significance in any of the cohorts (Figures 9c,f,i), patients who achieved objective responses (CR/PR) exhibited a tendency toward lower risk scores compared with those with non-response (SD/PD). Moreover, when comparing proportions, the low-risk group consistently showed a higher percentage of CR/PR cases: 23.39% vs. 22.05% in IMvigor210, 55% vs. 20% in GSE78220, and 30% vs. 17.95% in GSE91061 (Figures 9d,g,j). Although the differences did not reach statistical significance, a clear trend was observed in GSE78220 and GSE91061, with low-risk patients more likely to benefit from ICB therapy. Overall, these findings indicate that the immune-related risk score not only mirrors the underlying immune checkpoint expression landscape but also provides a clinically applicable biomarker for stratifying HNSCC patients who are more likely to benefit from ICB therapy.

### Analysis of drug sensitivity based on the IRG signature

To evaluate the therapeutic implications of the immune-related risk signature, we analyzed the correlation between risk score and predicted drug sensitivity across cancer cell lines. The present study focused on representative drugs for which higher risk scores were associated with significantly lower IC_50_ values, reflecting greater sensitivity in the high-risk group. Correlation analyses confirmed strong negative associations between risk score and IC_50_ values for Paclitaxel (R = −0.291, *p* = 0.0239), Tamoxifen (R = −0.4, *p* = 0.00154), PX-316 (R = −0.273, *p* = 0.0349), Carfilzomib (R = −0.35, *p* = 0.00617), and MK-8033 (R = −0.345, *p* = 0.00701) (Supplementary Figures S5a–e, left panels). Consistently, group comparisons showed that high-risk cell lines exhibited significantly lower IC_50_ values for all five agents compared with the low-risk group, indicating enhanced drug sensitivity (Supplementary Figures S5a–e, right panels). Such findings underscore the potential of this risk model to provide guidance for personalized treatment strategies and to inform the rational selection of therapeutic agents in HNSCC.

### Investigation of the expression and prognostic role of the PYGL gene in pan-cancer and HNSCC

**FIGURE 10:**
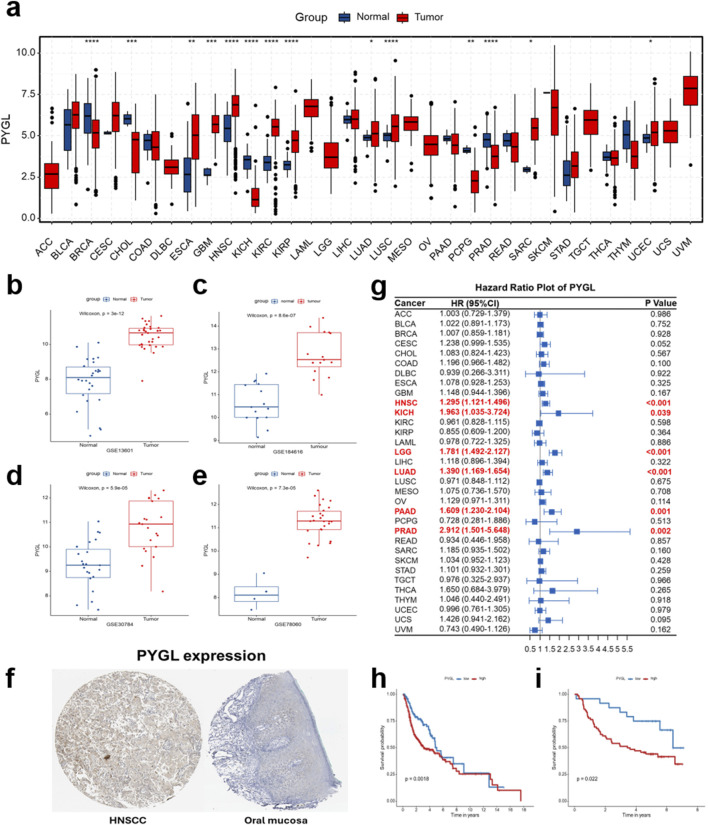
Expression and prognostic role of PYGL gene in pan-cancer. **(a)** Pan-cancer comparison of mRNA expression of the PYGL gene between tumor and normal tissues across multiple TCGA cancer types. **(b–e)** mRNA expression of PYGL in HNSCC and normal tissues from independent GEO datasets (GSE13601, GSE184616, GSE30784, and GSE78060). **(f)** Protein expression of PYGL in HNSCC and oral mucosa from the Human Protein Atlas (HPA) database. **(g)** Forest plot summarizing hazard ratio (HR) and 95% confidence interval (95%CI) for PYGL expression in relation to overall survival (OS) across various cancer types, with significant associations highlighted in red. **(h,i)** Kaplan-Meier survival analyses exhibiting the prognostic value of PYGL expression in the TCGA-HNSCC cohort **(h)** and the GSE41613 dataset **(i)**.

PYGL was prioritized for further investigation, as it showed the highest regression coefficient in our prognostic risk model, indicating its potential as a key driver gene within the signature. Therefore, we conducted further investigations focusing on its expression and prognostic role. To comprehensively evaluate PYGL, we first examined its mRNA levels across pan-cancer cohorts from TCGA. PYGL was significantly upregulated in multiple cancer types compared with matched normal tissues (Figure 10a). In the TCGA-HNSCC cohort, PYGL expression was markedly elevated in tumor samples relative to adjacent normal tissues, a finding further validated across four independent GEO datasets (GSE13601, GSE184616, GSE30784, and GSE78060), consistently demonstrating higher PYGL mRNA expression in HNSCC (Figures 10b–e). At the protein level, immunohistochemistry analysis based on the Human Protein Atlas (HPA) database revealed intense PYGL staining in HNSCC pathological tissues, whereas only weak or negligible expression was detected in normal oral mucosa (Figure 10f). These findings confirm the upregulation of PYGL in HNSCC at both the mRNA and protein levels.

We next assessed the prognostic value of PYGL expression. Pan-cancer survival analysis indicated that high PYGL expression was significantly correlated with poorer OS across several cancer types, with particularly strong associations in HNSCC (Figure 10g). Kaplan-Meier survival curves further confirmed that HNSCC patients with elevated PYGL expression experienced significantly worse outcomes, both in the TCGA-HNSCC and GSE41613 cohort (Figures 10h,i). These findings highlight *PYGL* as a critical oncogenic gene with strong prognostic implications, underscoring its potential as a biomarker for risk stratification and a promising candidate for future therapeutic exploration in HNSCC.

### Functional validation of the PYGL gene in HNSCC cell lines

**FIGURE 11:**
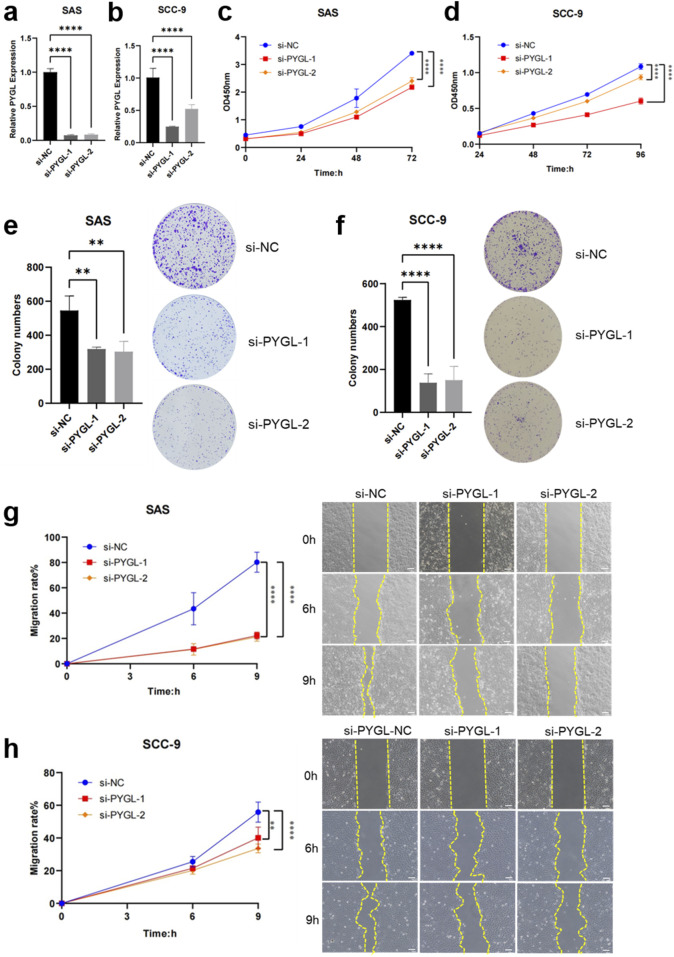
Knockdown of PYGL inhibits the proliferation and migration in HNSCC cell lines. **(a,b)** Knockdown efficiency of PYGL assessed by the quantitative real-time PCR (qRT-PCR) assay in SAS **(a)** and SCC-9 **(b)** cells. **(c,d)** Cell proliferation measured by CCK-8 assay at 0, 24, 48, and 72 h post-PYGL knockdown in SAS **(c)** and SCC-9 **(d)** cells. **(e,f)** Representative images and quantification of colony formation assays in SAS **(e)** and SCC-9 **(f)** cells following PGYL knockdown. **(g,h)** Wound healing assays showing migration rates in SAS **(g)** and SCC-9 **(h)** cells after PGYL knockdown. **p < 0.01, ****p < 0.0001.

Consideration that PYGL is highly expressed and associated with poor prognosis in HNSCC, we next performed *in vitro* experiments to elucidate its functional role. qRT-PCR confirmed that PYGL mRNA levels were efficiently silenced by two independent siRNAs (si-PYGL-1 and si-PYGL-2) in SAS and SCC-9 cells compared with the negative control (si-NC) (Figures 11a,b). CCK-8 assays revealed that PYGL knockdown significantly inhibited the proliferation of both SAS and SCC-9 cells in a time-dependent manner (Figures 11c,d). Consistently, colony formation assays demonstrated a marked reduction in the number of colonies following PYGL silencing, indicating impaired clonogenic capacity (Figures 11e,f). Furthermore, wound healing assays showed that suppression of PYGL expression significantly attenuated the migratory ability of SAS and SCC-9 cells, as evidenced by slower wound closure rates over time (Figures 11g,h). These functional experiments demonstrated that PYGL knockdown could inhibit the proliferation and migration of HNSCC cells, supporting its role as a potential oncogenic driver and therapeutic target.

### Analysis of transcriptomic alterations following PYGL silencing in HNSCC cells

**FIGURE 12:**
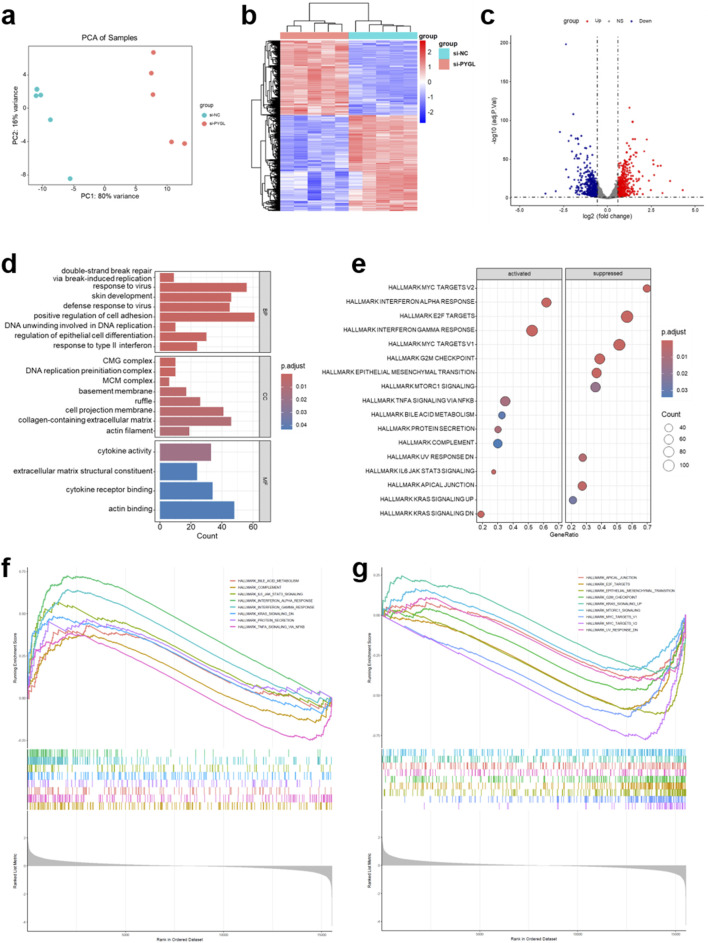
Transcriptomic analysis of PYGL knockdown in HNSCC cells. **(a)** Principal component analysis (PCA) comparing the PYGL knockdown and control groups. **(b)** Heatmap of hierarchical clustering showing differentially expressed genes (DEGs) between the two groups. **(c)** Volcano plot displaying DEGs, with significantly upregulated (red) and downregulated (blue) genes. **(d)** Bar plots of Gene Ontology (GO) enrichment analysis for DEGs. **(e)** Bubble plot of hallmark pathways significantly enriched in the PYGL knockdown groups based on GSEA. **(f,g)** Positively **(f)** and negatively **(g)** enriched key hallmark pathways in the PYGL knockdown groups based on GSEA.

Finally, we explored its downstream molecular mechanisms using transcriptome sequencing in SCC-9 cells after PYGL knockdown. Principal component analysis (PCA) revealed a clear separation between the si-NC and si-PYGL groups, indicating pronounced transcriptomic reprogramming upon PYGL silencing (Figure 12a). Consistently, hierarchical clustering analysis confirmed distinct expression profiles, with DEGs forming two major clusters that clearly distinguished the PYGL-silenced cells from controls (Figure 12b). A volcano plot was generated to visualize the transcriptional changes, with 545 genes upregulated and 697 genes downregulated (Figure 12c). To gain functional insights, GO analysis was performed, which demonstrated that DEGs were clustered into several functional categories, including immune response, cell adhesion, DNA replication-related processes, and extracellular matrix organization (Figure 12d). GSEA revealed both significantly activated and suppressed hallmark gene sets related to immune response, cell adhesion, and migration (Figure 12e). PYGL knockdown markedly activated pathways involved in immune activation, inflammatory signaling, and metabolic adaptation, including bile acid metabolism, complement cascade, IL6/JAK/STAT3 signaling, and interferon responses, suggesting that PYGL normally suppresses these processes (Figure 12f). In contrast, PYGL knockdown inhibited pathways associated with proliferation, cell cycle progression, and metastasis, such as the G2/M checkpoint, mTORC1 signaling, epithelial–mesenchymal transition, and MYC target gene programs, indicating that PYGL normally promotes tumor growth and progression (Figure 12g). These transcriptomic alterations suggest that PYGL plays a dual role in HNSCC by promoting proliferative and metastatic programs while restraining immune and inflammatory responses.

## Discussion

Despite therapeutic advancements, the prognosis for patients with HNSCC remains dismal, particularly for those diagnosed at advanced stages. The complexity of HNSCC necessitates a deeper understanding of its biological underpinnings, especially the TME, which plays a crucial role in tumor progression and therapeutic resistance (Vyhnankova et al., 2025). However, the molecular drivers of the immune-hot and immune-cold phenotypes, and how they collectively influence prognosis and therapy response, remain incompletely characterized. In this study, we focused on characterizing the immune cell infiltration patterns within the TME of HNSCC, specifically distinguishing between “hot” and “cold” tumors. Our findings revealed significant discrepancies in immune cell presence and activity, which are pivotal in determining therapeutic outcomes. We constructed and validated a prognostic signature based on IRGs, providing insights into the implications of immune infiltration on patient survival and treatment response.

Immune-hot tumors demonstrated pronounced infiltration by activated CD8^+^ T cells and activated B cells, which are widely recognized as mediators of effective antitumor immunity and favorable prognosis in multiple solid tumors (Xie et al., 2025; Zhang L. et al., 2024). This enrichment of effector lymphocytes is mechanistically linked to the upregulation of chemokines such as CXCL9 and CXCL10, which facilitate T cell recruitment, a phenomenon also observed in HNSCC subtypes with enhanced immune responsiveness (Chen et al., 2023). In contrast, immune-cold tumors exhibited reduced effector cell infiltration, likely due to the activation of immunosuppressive pathways, including TGF-β and WNT signaling, which have been shown to exclude T cells from the tumor parenchyma and promote immune evasion (Zhang et al., 2020). Moreover, the association between low immune infiltration and poor survival aligned with previous computational and pathological studies, which revealed that diminished CD8^+^ T cells and memory CD4^+^ T cells correlated with adverse clinical outcomes (Zhang L. et al., 2024; Jin and Qin, 2020). Notably, our functional enrichment analyses further support these observations by highlighting negative enrichment of immune activation pathways and response to chemokine in cold tumors, suggesting a microenvironmental shift toward immunological quiescence. Thus, the immune-cell infiltration landscape in HNSCC is shaped by a complex interplay of chemokine expression and immunosuppressive signaling, providing a mechanistic basis for the divergent clinical trajectories of immune-hot and immune-cold tumors.

Next, based on the DEGs between the immune infiltration-related clusters, we employed a series of rigorous selection strategies to develop a six-gene immune-related gene signature. The signature effectively stratified HNSCC patients into low- and high-risk groups, demonstrating robust predictive accuracy for 1-, 3-, and 5-year OS, and the reliability and predictive accuracy were further confirmed by internal and external validation cohorts. This aligned with recent studies emphasizing the prognostic utility of immune-based signatures in HNSCC. For instance, Wang et al. constructed a 9-immune-gene signature that significantly discriminated risk groups and independently predicted survival outcomes in early-stage HNSCC patients (Wang et al., 2024). Similarly, Batsaki et al. identified a 5-gene immune signature derived from peripheral blood, which correlated with poor prognosis and highlighted the potential of non-invasive biomarkers for risk assessment in HNSCC (Batsaki et al., 2025). Notably, single-cell transcriptomic analyses elucidated distinct expression patterns of these genes across immune and malignant cell compartments, suggesting context-dependent regulatory roles that may modulate immune cell recruitment, activation, or suppression (Chen X. et al., 2025).

While prior work established IRG signatures for prognosis, our integration of gene expression with mutation burden and immune infiltration confers enhanced predictive resolution, especially when contrasted with earlier models that did not account for the spatial or functional heterogeneity of the tumor immune landscape (Zhu et al., 2022). The differential mutation profiles between distinct risk groups, coupled with higher TMB in high-risk patients, provide mechanistic insights into the poor prognosis associated with this subgroup. Specifically, elevated TMB in high-risk patients was associated with worse survival, suggesting increased genomic instability and potential immune evasion mechanisms. This result was consistent with findings by Sacconi et al., who reported that immune gene signatures in HNSCC are influenced by *TP53* mutation status and co-mutations, where specific mutational patterns correlate with enhanced immune gene expression and altered clinical outcomes (Sacconi et al., 2023). Moreover, Ma et al. demonstrated that tumor-infiltrating immune-related signatures, including those involving non-coding RNAs, can reflect mutational landscapes and predict responses to immunotherapy, such as PD-1 blockade, which may be relevant to TMB-driven immune activation (Ma et al., 2021). Critically, combining TMB with our IRG risk score yielded superior prognostic stratification: patients with low-risk/low-TMB exhibited the best survival, whereas those with high-risk/high-TMB had the worst outcomes, suggesting that a favorable prognosis requires both a high mutational burden and a permissive immune microenvironment. This synergy aligns with emerging paradigms in immuno-oncology, where both antigenicity (TMB) and immune competence (IRG signature) are required for effective anti-tumor immunity (Sacconi et al., 2023; Meehan et al., 2020). Our IRG signature not only serves as a reliable prognostic tool but also, when combined with TMB, enhances patient stratification for personalized management. Further mechanistic insights were gleaned from the differential immune cell infiltration and immune checkpoint expression between high- and low-risk groups. Our data demonstrated that patients in the low-risk group harbored higher levels of effector immune cells, including activated T cells and effector memory T cells, and concomitantly expressed elevated levels of immune checkpoint molecules such as PD-1, CTLA-4, and TIGIT, markers associated with both immune activation and the potential for checkpoint blockade efficacy (Lu et al., 2021; Ar et al., 2023). While these patterns mirrored those identified in prior immune-infiltration studies in HNSCC (Zhang L. et al., 2024; Ruhle et al., 2022; Hoffmann et al., 2023), our work uniquely establishes a strong correlation between risk score, immune cell composition, and checkpoint expression, suggesting a mechanistic basis for the observed clinical heterogeneity in immunotherapeutic response. Future studies should validate this model in larger, multi-center cohorts and explore its utility in guiding immunotherapy decisions, building on the growing evidence that immune and mutational profiling are integral to advancing HNSCC care.

Finally, PYGL was prioritized for further investigation due to its highest regression coefficient in the prognostic risk model, suggesting its potential as a key driver within the signature. Our functional and transcriptomic analyses collectively establish PYGL as a central metabolic regulator in HNSCC, orchestrating not only energy metabolism but also critical oncogenic signaling and immunomodulatory programs. While our phenotypic assays confirmed that PYGL knockdown significantly impairs tumor cell proliferation, colony formation, and migratory capacity, consistent with its role in fueling glycolysis and supporting biosynthetic demands under metabolic stress (Ji et al., 2023; Guan et al., 2023), transcriptomic profiling provides unprecedented mechanistic depth into how PYGL loss reprograms the cellular landscape. GO analysis further revealed that PYGL depletion led to significant downregulation of biological processes related to DNA replication, cell cycle progression, and extracellular matrix (ECM) organization, including “DNA unwinding involved in DNA replication”, “MCM complex”, and “collagen-containing extracellular matrix”. These findings are highly consistent with PYGL’s known role in sustaining nucleotide synthesis and promoting EMT via ECM remodeling enzymes such as MMPs (Ji et al., 2023). The downregulation of gene sets associated with “actin filament” and “cell projection membrane” terms further supports impaired cytoskeletal dynamics and cell motility, aligning with the observed reduction in migration following PYGL knockdown. More importantly, GSEA using hallmark gene sets uncovered a striking dichotomy: while pro-oncogenic pathways were suppressed, immune- and stress-responsive pathways were activated upon PYGL loss. Specifically, hallmark gene sets such as HALLMARK_MYC_TARGETS_V1/V2, HALLMARK_E2F_TARGETS, HALLMARK_G2M_CHECKPOINT, and HALLMARK_EPITHELIAL_MESENCHYMAL_TRANSITION were significantly suppressed, directly linking PYGL activity to core drivers of proliferation, cell cycle progression, and metastasis (Ji et al., 2023; Cao and Wang, 2024). Conversely, PYGL knockdown activated key immune-related and stress-response hallmarks, including HALLMARK_INTERFERON_ALPHA/GAMMA_RESPONSE, HALLMARK_IL6_JAK_STAT3_SIGNALING, and HALLMARK_PROTEIN_SECRETION. These results suggest that inhibiting PYGL may reverse immunosuppressive features of the TME, which has profound therapeutic implications. Recent studies have shown that high PYGL expression is correlated with reduced CD8^+^ T-cell infiltration and upregulated immune checkpoint molecules in HNSCC, contributing to immune evasion (Chen X. et al., 2025; Jiang et al., 2025). Collectively, our data position PYGL as a potential nexus linking glycogen metabolism to oncogenic signaling and immune evasion in HNSCC. Its inhibition not only suppresses tumor-intrinsic proliferative and migratory programs but also transcriptionally reprograms the TME toward an immunostimulatory state, a dual effect that warrants further preclinical investigation.

Despite the robustness of our findings across multiple public cohorts, several limitations warrant acknowledgment. First, the retrospective nature and absence of prospectively collected, treatment-annotated clinical samples limit the systematic assessment of the signature’s predictive value for immunotherapy. Future studies should prioritize more rigorous validation in treatment-annotated cohorts and highlight the utility of integrating single-cell RNA sequencing with bulk transcriptomics to deconvolute the tumor immune microenvironment with higher precision (Lai et al., 2025). Second, our model relies on conventional single-gene expression, which may be susceptible to batch effects across platforms. Advanced modeling frameworks, such as gene-pair ratios, have demonstrated enhanced robustness and generalizability (Xie et al., 2022), and adopting such approaches represents a valuable direction for future improvement. Third, patient stratification in this study depends on cohort-specific cut-off values (e.g., median risk score), which significantly limits external validity and direct clinical application. To enhance translatability, future work should establish universally applicable cut-offs using large, multi-center cohorts or employ a continuous risk score system (Zhang et al., 2022). Finally, while we performed functional validation for PYGL, the study primarily establishes statistical associations between the signature genes and prognosis. Future studies should employ causal inference methods, such as Mendelian Randomization (Zhang C. et al., 2024), alongside detailed mechanistic experiments to distinguish causal drivers from passive correlates.

## Conclusion

In summary, this research delineates the distinct TME characteristics between “cold” and “hot” tumors in HNSCC and underscores the prognostic value of IRGs. The findings not only contribute to the understanding of immune response variations but also highlight potential avenues for refining immunotherapy strategies. Future studies should aim to validate these prognostic markers in larger, multi-center cohorts and explore innovative approaches to enhance immune responses, particularly in cold tumors, thereby improving treatment outcomes for HNSCC patients.

## Data Availability

The public datasets presented in this study can be found in online repositories. The names of the repository/repositories and accession number(s) can be found in the article. The raw RNA-seq data of PYGL knockdown have been deposited in the NCBI BioProject database (accession number PRJNA1356554) (https://www.ncbi.nlm.nih.gov/bioproject/PRJNA1356554).
