# Supplementary material for: A novel immune-related gene signature stratifies prognosis and characterizes the tumor immune microenvironment in head and neck squamous cell carcinoma
Source: Front Cell Dev Biol. 2026 Apr 8;14:1756922. doi: 10.3389/fcell.2026.1756922 (PMC13099764; doi:10.3389/fcell.2026.1756922)
Supplement: Supplementary file 5 [file DataSheet1.docx]

**
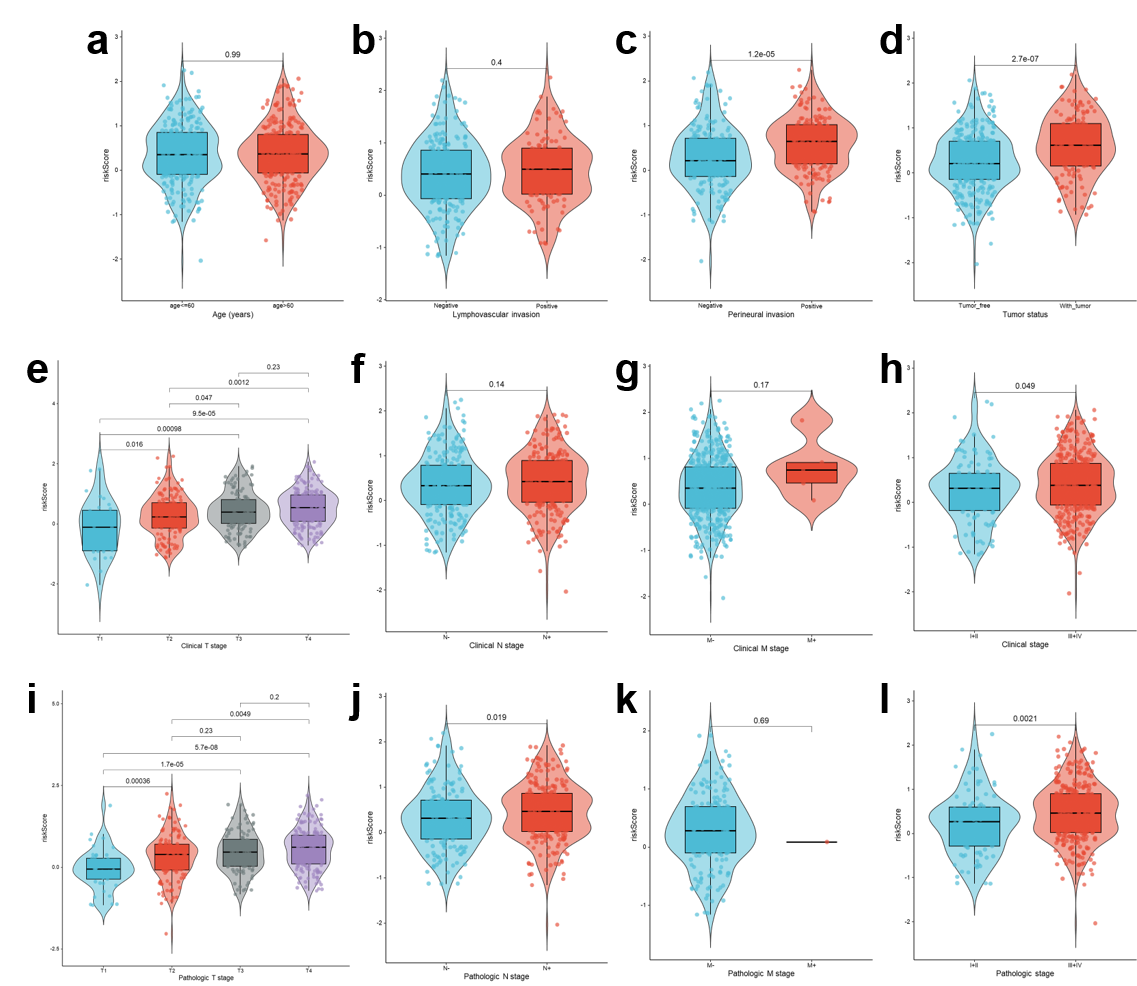
**

**Figure S1. Association of IRG signature-derived risk scores with** **clinicopathological characteristics in the TCGA-HNSCC cohort.**

(a-l) Violin plots depicting the distribution of risk scores across subgroups defined by various clinicopathological characteristics in HNSCC patients.


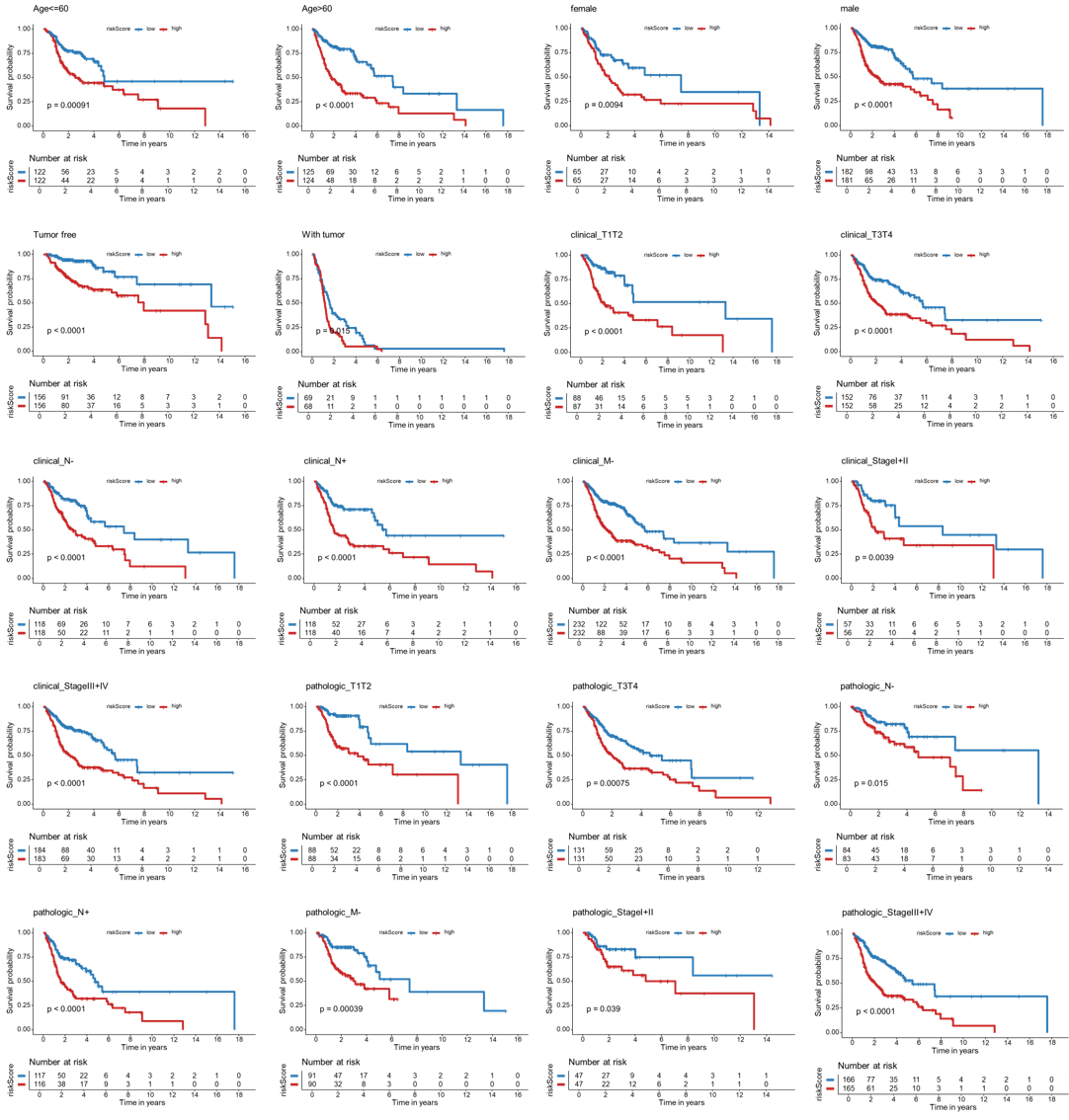


**Figure S2. Kaplan-Meier subgroup survival analyses of TCGA-HNSCC patients stratified by the IRG signature.**


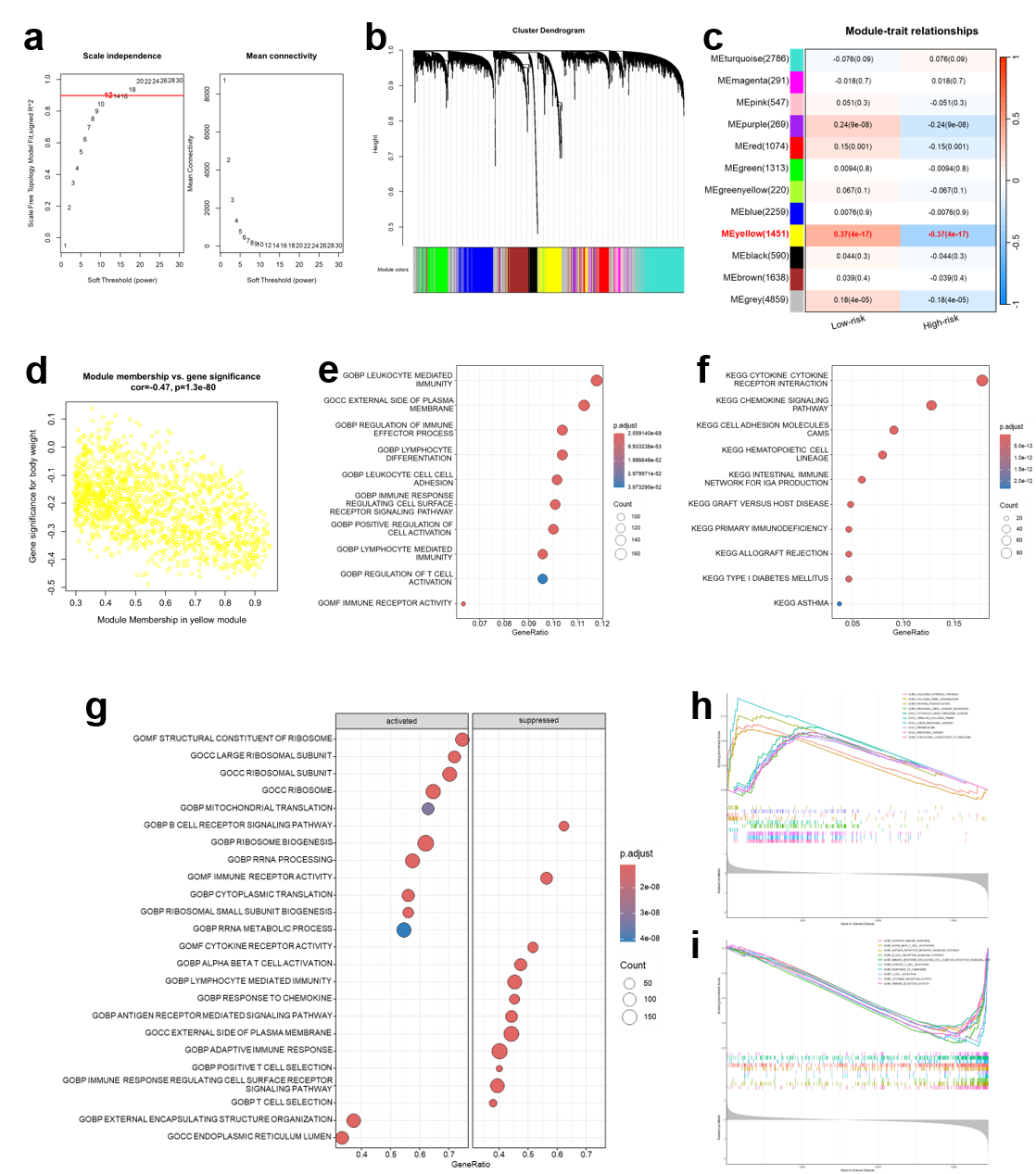


**Figure S3. WGCNA and GSEA** **of the IRG signature.**

(a) Evaluation of scale independence (left) and mean connectivity (right) for different soft thresholding powers in WGCNA for the TCGA-HNASCC cohort. The red line indicates the optimal power value. (b) Clustering dendrogram of genes, with different colors representing distinct co-expression modules identified by WGCNA in the TCGA-HNASCC cohort. (c) Heatmap showing the correlation between each gene module and risk groups. (d) Correlation analysis between module membership and gene significance in the yellow module. (e, f) Gene Ontology (GO) (e) and Kyoto Encyclopedia of Genes and Genomes (KEGG) (f) enrichment analyses of genes in the yellow module. (g) Bubble plot of significantly enriched GO pathways in the high-risk groups based on GSEA. Enrichment plots of the top 10 GO pathways activated (h) and suppressed (i) in the high-risk group.


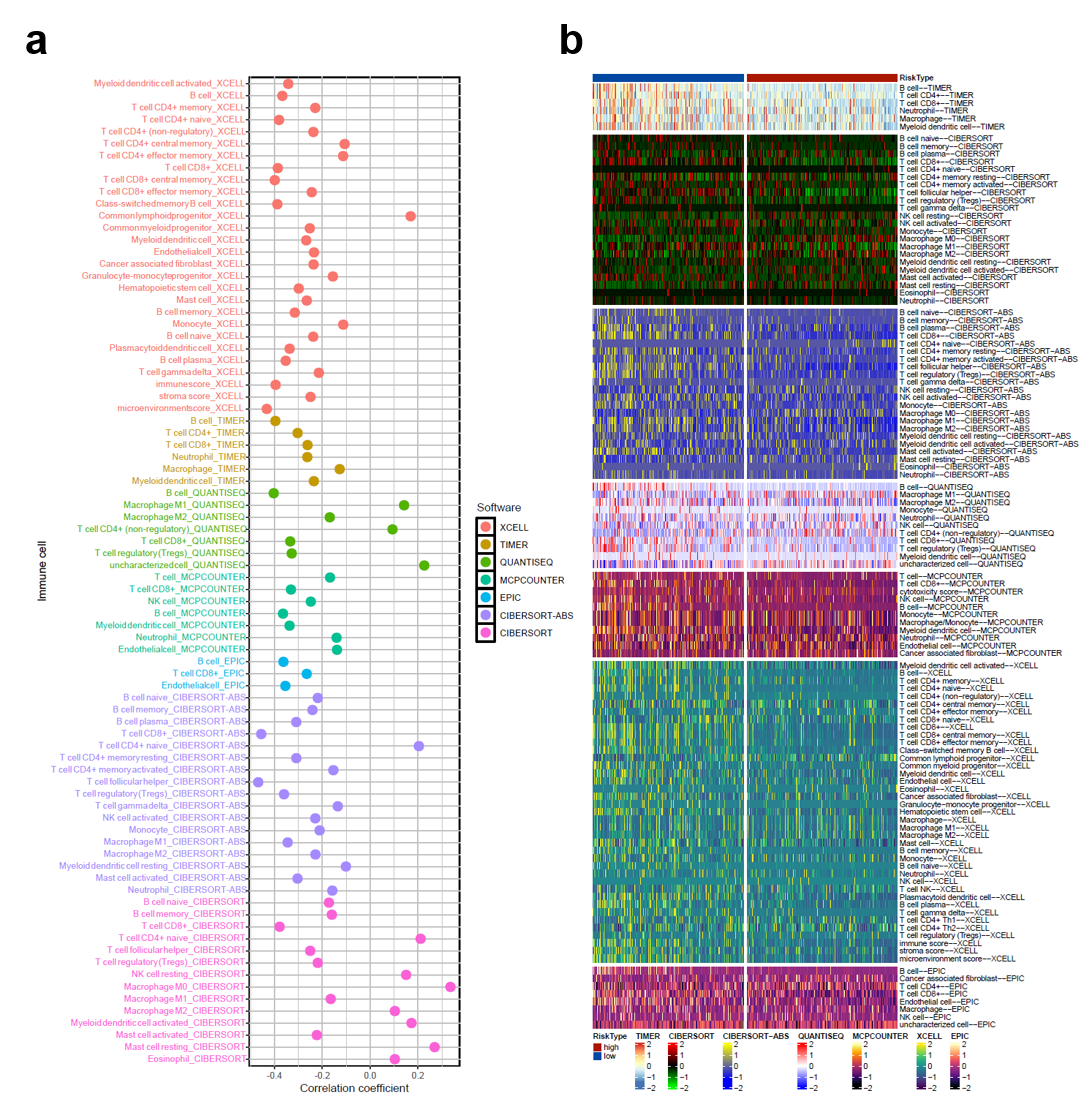


**Figure S4. Validation of immune infiltration patterns using multiple deconvolution algorithms.**

(a) Correlation analysis between risk score and the abundance of immune cell subtypes across different deconvolution algorithms (CIBERSORT, TIMER, xCell, EPIC, MCP-counter, and quanTIseq). (b) Heatmaps illustrating the relative abundance of immune cell subsets in low-risk (blue) versus high-risk (red) HNSCC patients, as estimated by each of the deconvolution algorithms.


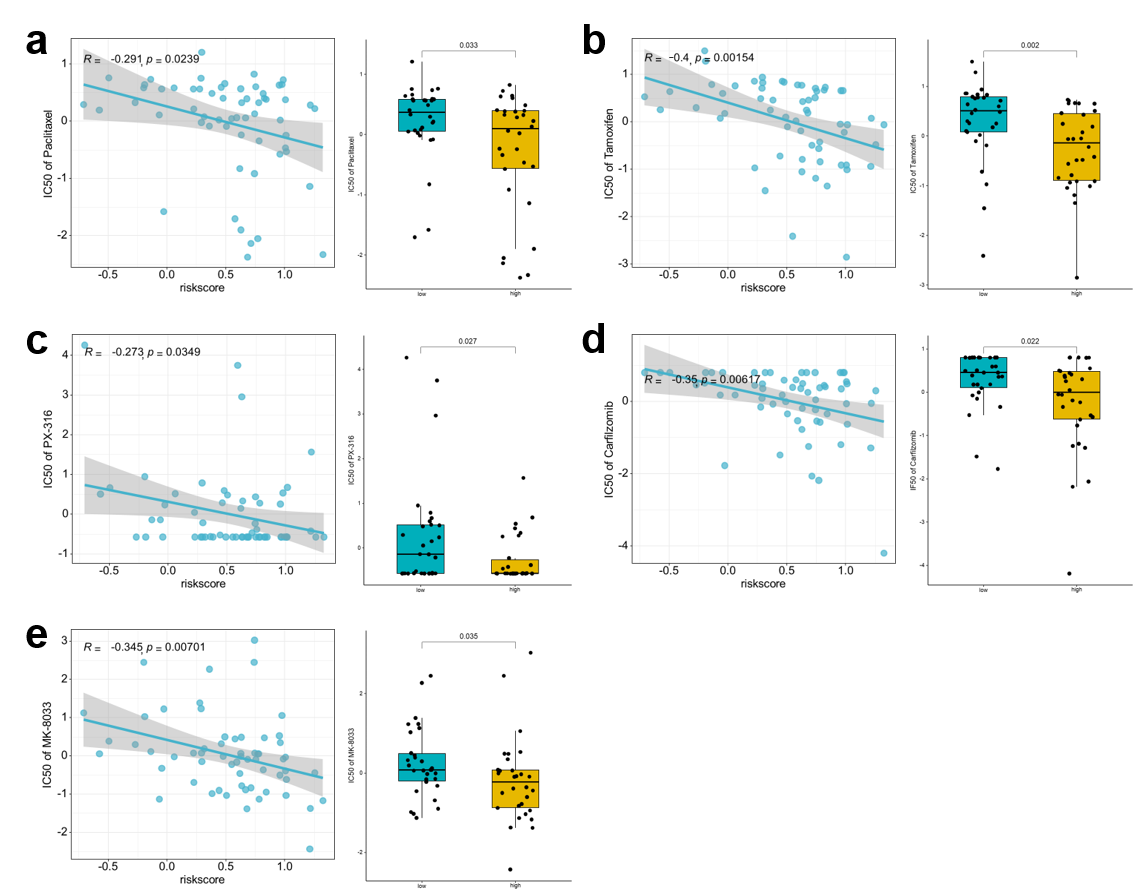


**Figure S5. Correlation between the IRG signature and drug sensitivity.**

(a-e) Pearson correlation analysis between risk score and drug sensitivity for multiple agents (left panel). Comparison of estimated half-maximal inhibitory concentration (IC_50_) values for these agents between low- and high-risk groups defined by the signature (right panel).
